# Efferocytic Defects in Early Atherosclerosis Are Driven by GATA2 Overexpression in Macrophages

**DOI:** 10.3389/fimmu.2020.594136

**Published:** 2020-10-23

**Authors:** Charles Yin, Angela M. Vrieze, Mara Rosoga, James Akingbasote, Emily N. Pawlak, Rajesh Abraham Jacob, Jonathan Hu, Neha Sharma, Jimmy D. Dikeakos, Lillian Barra, A. Dave Nagpal, Bryan Heit

**Affiliations:** ^1^Department of Microbiology and Immunology, and The Center for Human Immunology, The University of Western Ontario, London, ON, Canada; ^2^Division of Rheumatology, Department of Medicine, The University of Western Ontario, London, ON, Canada; ^3^Division of Cardiac Surgery, Department of Surgery, The University of Western Ontario, London, ON, Canada; ^4^Division of Critical Care Medicine, Department of Medicine, The University of Western Ontario, London, ON, Canada; ^5^Robarts Research Institute, London, ON, Canada

**Keywords:** macrophage, atherosclerosis, inflammation, GATA2, phagocytosis, efferocytosis, vesicular trafficking

## Abstract

The loss of efferocytosis—the phagocytic clearance of apoptotic cells—is an initiating event in atherosclerotic plaque formation. While the loss of macrophage efferocytosis is a prerequisite for advanced plaque formation, the transcriptional and cellular events in the pre-lesion site that drive these defects are poorly defined. Transcriptomic analysis of macrophages recovered from early-stage human atherosclerotic lesions identified a 50-fold increase in the expression of GATA2, a transcription factor whose expression is normally restricted to the hematopoietic compartment. GATA2 overexpression *in vitro* recapitulated many of the functional defects reported in patient macrophages, including deficits at multiple stages in the efferocytic process. These findings included defects in the uptake of apoptotic cells, efferosome maturation, and in phagolysosome function. These efferocytic defects were a product of GATA2-driven alterations in the expression of key regulatory proteins, including Src-family kinases, Rab7 and components of both the vacuolar ATPase and NADPH oxidase complexes. In summary, these data identify a mechanism by which efferocytic capacity is lost in the early stages of plaque formation, thus setting the stage for the accumulation of uncleared apoptotic cells that comprise the bulk of atherosclerotic plaques.

## Introduction

Macrophages are the primary immune cell type driving the initiation and progression of atherosclerosis. Under homeostatic conditions macrophages are atheroprotective, through both the endocytic clearance of lipoprotein deposits and through efferocytosis—the phagocytic clearance of apoptotic cells. Combined, these processes prevent the accumulation of lipids and apoptotic cells, as well as induce anti-inflammatory signaling, thereby countering the major pathological processes required for plaque formation (1–4). Although macrophages are normally capable of processing and exporting cholesterol in an efficient manner (5, 6), during atherosclerosis the burden of low-density lipoprotein (LDL) and its chemically modified variants such as oxidized LDL (oxLDL) exceed the macrophages’ processing capacity, resulting in the accumulation of intracellular cholesterol and the pathological differentiation of these macrophages into lipid-laden foam cells (7–10). In response to the cellular stress associated with cholesterol accumulation, foam cells secrete pro-inflammatory cytokines, undergo apoptosis, and are cleared by neighbouring macrophages through efferocytosis (11, 12). In parallel, oxLDL signaling through CD36, Toll-like receptor (TLR) 2 and TLR4 on macrophages and other plaque-resident immune cells further exacerbates inflammation (13, 14). Critically, as atherosclerosis progresses, macrophage efferocytosis within the lesion becomes defective, leaving apoptotic foam cells uncleared. These apoptotic cells eventually undergo secondary necrosis, releasing pro-inflammatory alarmins and generating the necrotic core of the plaque (15–17). The resulting inflammatory plaque microenvironment induces the recruitment of inflammatory (Ly6C^high^) monocytes which first differentiate into inflammatory M1-polarized macrophages, and then into foam cells (18, 19). Lysosomal proteins released during necrosis further destabilize the plaque, contributing to plaque rupture and exposure of thrombogenic factors contained within the plaque, resulting in thrombus formation that may lead to a stroke or myocardial infarct (15, 20, 21).

Macrophages are phenotypically plastic cells whose functional capabilities are determined by their polarization into specialized sub-types. These polarization states are determined by a combination of macrophage ontology (22, 23), tissue-specific cues (24), host age (25), and environmental cues (26). Macrophage polarization is tightly entwined with the development of atherosclerosis, but while multiple macrophage polarization states have been identified in mouse and human plaques, little is known about the transcription factors and the tissue- and environment-specific cues which regulate their transcriptional programs. In mice, resident aortic macrophages are embryonically-derived, self-renewing cells characterized by the expression of scavenger receptors, MHC II and efferocytic receptors (22). In mouse models of atherosclerosis, two macrophage sub-types emerge within atherosclerotic lesions: haematopoietically-derived inflammatory (M1) macrophages, and a TREM2^+^ population unique to the plaque microenvironment that express a mixture of resident-macrophage markers and markers of alternatively activated (M2) macrophages (27). Later in murine models of disease, Mox macrophages arise, accounting for 30% of plaque-resident macrophages, and express genes that regulate angiogenesis, anti-oxidant responses, and heme processing (28). Mox macrophages represent one of the only plaque-resident cell types with an established driving transcription factor—Nrf2 (29). While the ontology and transcriptional landscape of human plaque macrophages are not as well understood, macrophages with markers of M1-polarization (iNOS, CD86), M2-polarization (MR, dectin-1), and TREM2^+^-polarization (TREM2, CD9^high^) have been identified in established plaques (27, 30). In advanced stages of disease, other macrophage sub-types may emerge. For example, intraplaque hemorrhage in humans gives rise to the atheroprotective Mhem macrophage sub-type, which are resistant to oxidative stress and scavenge hemoglobin (31, 32). However, despite the identification of multiple macrophage subtypes within the atherosclerotic plaque, the transcription factors which drive these polarization states remain largely unknown, and the transcriptional programs that link microenvironmental cues to distinct polarization states in atherosclerotic macrophages remain only partially understood.

Most investigations of macrophage gene expression in atherosclerosis have concentrated on the stages of disease following the formation of advanced-stage plaques bearing a necrotic core and fibrous cap (33). As such, little is known of the transcriptional events which initiate disease in pre-lesion sites. During the early stages of disease, mouse models have demonstrated that lipid accumulation and endothelial activation drives inflammatory monocyte recruitment and differentiation into M1 macrophages (34). Local proliferation of these monocyte-derived macrophages further populates the plaque with these inflammatory cells (35). *In vitro* analyses of changes in macrophage gene expression following exposure to atherogenic lipids provides some insight into the transcriptional changes that occur early in disease. Koller *et al*. demonstrated that exposure of RAW264.7 murine macrophages to oxidized lipids resulted in the rapid upregulation of the genes involved in oxLDL uptake, apoptosis, and cell stress, while genes controlling cholesterol efflux and cell proliferation were downregulated (36). Using human monocyte-derived macrophages, Ho & Fraiser observed a similar trend following exposure to modified LDL (moLDL), and determined that complement protein C1q accelerates this response (37). However, these studies were limited to hematogenous macrophages cultured *in vitro*, and therefore lacked the full range of ontological, tissue-specific and environmental signals which drive macrophage transcriptional profiles *in vivo*.

In this study we investigated the transcriptional profile of macrophages recovered from early human aortic plaque, identifying a novel transcriptional state intermediary between non-polarized (M0) macrophages and inflammatory M1-like cells. This transcriptional state was characterized by the high expression of the hematopoietic transcription factor GATA2, with GATA2 overexpression *in vitro* generating efferocytic defects similar to those reported in atherosclerosis (38, 39). These macrophages downregulated several signaling molecules and vesicular trafficking regulators required for efferocytosis, and consequently, GATA2 overexpression resulted in impaired efferocytosis and efferosome maturation. Taken together, our data implicate GATA2 overexpression as a cause of efferocytic defects during the earliest stages of atherosclerotic plaque development in humans.

## Materials and Methods

### Materials

pBabePuro-GATA2 (Addgene #1285) was a gift from Gokhan Hotamisligil (40). Coverslips, slides, Leiden chambers and 16% paraformaldehyde (PFA) were from Electron Microscopy Sciences (Hatfield, PA). DMEM, RPMI and fetal bovine serum (FBS) were from Wisent (Montreal, Canada). Restriction enzymes, Gibson assembly reagent, and ligase were from New England Biolabs (Whitby, Canada). M-CSF, GM-CSF, INFγ, and IL-4 were purchased from Peprotech (Montreal, Canada). Micro-beads were from Bangs Laboratories (Fishers, Indiana). Lipids and cholesterol were from Avanti polar lipids (Alabaster, AL). THP1 cells, Lympholyte-poly, and all secondary antibodies/Fab’s were from Cedarlane Labs (Burlington, Canada). pHrodo, NBT, human oxLDL, TRITC-Dextran, nigericin, Hoechst 33342, NanoDrop 1000 Spectrophotometer, Fast SYBR Green Master Mix, QuantStudio 5 Real-Time PCR System, and anti-citrulline (modified) detection kit were from ThermoFisher Canada (Mississauga, ON). RNease RNA extraction kits were from Qiagen (Toronto, Canada). Human IgG, PMA, and anti-modified citrulline antibody were from Sigma-Aldrich (Oakville, ON). Anti-CD163, anti-GATA2, and anti-CD68 were from Abcam (Toronto, Canada). 4.0 mm CleanCut RCL Aortic Punch was from QUEST Medical (Allen, Texas). Gemini Fluorescence Microplate Reader running SoftMax Pro was from Molecular Devices (San Jose, California). PureZOL RNA isolation reagent and iScript Select cDNA Synthesis Kit were from BioRad (Hercules, California). CapSure HS LCM Caps and GeneChip WT Pico Reagent Kit were from Applied Biosystems (Forest City, California). Prism 6 software was from GraphPad (La Jolla, California). LDLR^-/-^ (B6.129S7-Ldlrtm1Her/J) mice were purchased from The Jackson Laboratory (Bar Harbor, Maine), and TD.150834 chow was purchased from Envigo (Indianapolis, Indiana). FIJI was downloaded from https://fiji.sc/ (41). All other materials were purchased from Bioshop Canada (Burlington, Canada).

### Human Plaque Histology and Macrophage Isolation

Patient tissues used in this study were obtained under a discarded tissue protocol from patients undergoing elective coronary artery bypass graft surgery at London Health Sciences Centre, London, Canada, that was approved by the Office of Human Research Ethics at Western University Health Sciences Research Ethics Board (HSREB Reference Number: 107566). All procedures were performed in accordance with the guidelines of the Tri-Council policy statement on human research. Since this is a discarded tissue study, we did not collect any personal health information, personal identifying information or clinical data other than the age range and the male-to-female ratio of the entire cohort. Researchers were blinded to patient identification and clinical characteristics. Aortic punch tissue specimens were obtained intra-operatively by a cardiac surgeon using a 4.0-mm diameter aortic punch and placed in cold saline. Specimens were then bisected evenly using clean surgical scissors, with one half of the tissue used to prepare frozen sections and the other for preparing paraffin sections. For laser capture microdissection, tissue specimens were embedded in OCT freezing compound and frozen on dry ice over a period of 5–10 min within 30 min of collection, then placed into -80°C for storage. For immunofluorescence and Oil-Red-O (ORO) staining, tissue specimens were fixed in fresh 4% paraformaldehyde (PFA) for 24 h at 4°C, placed in PBS + 15% sucrose until the tissue lost buoyancy (~4 h), and then placed into PBS + 30% sucrose overnight at 4°C. For all other stains, paraffin-embedded sections were fixed 4% PFA for 24 h at 4°C, then stored in 70% ethanol. Specimens were dehydrated by 1 h immersion in 70% and 95% ethanol, followed by four immersions in 100% ethanol (1, 1.5, 1.5, and 2 h). Sections were cleared with two 1 h immersions in xylene, then immersed 2 times 1 h in paraffin wax (58°C). Processed tissues were embedded into paraffin blocks.

OCT-embedded tissues were sectioned into 10 μm sections and paraffin-embedded samples at 5 μm, and placed onto clean, RNAse-free slides. All histology (H&E, ORO, Movat’s pentachrome stain and TUNEL stain) was performed at the Robarts Molecular Pathology core.

For laser capture microdissection (LCM), slides were stored at -80C until processing. Slides were fixed in ice-cold acetone for 2 min, air-dried for 30 s and stained with primary antibody (anti-CD163, 30–60 µg/ml, 3 min), washed 2× with PBS, and stained with a secondary antibody (1:100 dilution, 3 min). CD163 was used in lieu of CD68, as CD163 is found on all human macrophages where as CD68 stains cells in the atheroma of both macrophage and smooth muscle origin (42). After two PBS washes, the slides were dehydrated by sequential addition of 75%, 95%, and 100% ethanol (30 s/step) and dried by immersion in xylene for 5 min. LCM was performed on an ArcturusXT Laser Capture Microdissection System (Applied Biosystems) using CapSure HS LCM Caps. Samples were captured using IR capture laser of 15 µm diameter at 70 mW, 1,500 µs and 1 hit per capture. A minimum of 200 captures per section across four consecutive sections was performed to obtain sufficient cell numbers for downstream analysis.

### Microscopy and Image Analysis

Unless otherwise noted, all microscopy was performed using a Leica DMI6000B microscope equipped with 40×/1.40NA, 63×/1.40NA, and 100×/1.40 NA objectives, photometrics Evolve-512 delta EM-CCD camera, heated/CO_2_ perfused stage, Chroma Sedat Quad filter set with blue (Ex: 380/30, Em: 455/50), green (Ex: 490/20, Em: 525/36), red (Ex: 555/25, Em: 605/52), and far-red (Ex: 645/30, Em: 705/72) filter pairs, and the LAS-X software platform. All image analysis was performed in FIJI (41). For quantitative imaging, the same exposure intensity, exposure time and EM-CCD gain setters were used within a single experiment, and control-normalized data used for comparisons between experiments.

### Primary Macrophage Culture

The collection of blood from healthy donors was approved by the Health Science Research Ethics Board of the University of Western Ontario and was performed in accordance with the guidelines of the Tri-Council policy statement on human research. Blood was drawn from age- and sex-matched volunteers without diagnosed coronary artery disease by venipuncture. Blood was collected with heparinized tubes, 5 ml of blood layered over 5 ml of Lympholyte-poly, and centrifuged at 300 × g for 35 min. The top band of cells was collected, washed once (300 × g, 6 min, 20°C) with phosphate buffered saline (PBS, 137 mM NaCl, 10 mM Na_2_HPO_4_), and resuspended at 2 × 10^6^ cells/ml in RPMI-1640 + 10% FBS + 1% antibiotic–antimycotic. 200 μl of this suspension was placed on sterile glass coverslips for 1 h at 37°C, washed twice with PBS, and then differentiated into M0-, M1-, or M2-polarized macrophages as per our published protocols (43, 44). Briefly, M0-polarization was achieved by incubation of freshly isolated human monocytes with 10 ng/ml recombinant human M-CSF for 5–7 days. M1-polarization was achieved by culture with 10 ng/ml recombinant human M-CSF for 5 days and then 20 ng/ml recombinant human GM-CSF, 250 ng/ml LPS and 10 ng/ml IFN-γ for an additional 2 days. M2-polarization was achieved by culture with 10 ng/ml recombinant human M-CSF for 5 days and then 10 ng/ml recombinant human M-CSF and 10 ng/ml IL-4 for an additional 2 days.

### Microarray

Total RNA was prepared from patient aortic punch macrophages or M0-polarized control macrophages by TRIzol extraction. RNA quality was checked using a 2100 Bioanalyzer Instrument (Agilent, Santa Clara, California), with samples below an A260/280 of 1.5 and/or RIN of seven rejected from analysis. A minimum of 2.0 µg of total RNA from each sample was used in the microarray. RNA samples were prepared for array hybridization using the GeneChip WT Pico Reagent Kit according to the manufacturer’s instructions with 12 cycles of pre-*in vitro* transcription amplification. Poly-A RNA standards provided by the manufacturer were used as exogenous positive controls. Samples were analyzed on the GeneChip Human Gene 2.0 ST Array chip hybridized on the GeneChip Scanner 3000 7G System (Applied Biosystems) according to the manufacturer’s recommended instructions. Raw microarray data were analyzed using the Partek Genomics Suite platform (Partek), with reads normalized by a Robust Multi-array Average (RMA) procedure with quantile normalization. Mixed-model ANOVA was used to identify differentially expressed genes with a fold-change cutoff of 2.0 and p-value cutoff of p<0.05. Unsupervised hierarchical clustering of all differentially expressed genes was performed to generate a heat map of differential gene expression. Results were verified using Bioconductor (45). The oligo package was used to read in raw microarray data files and perform RMA normalization, and the limma package used to identify differentially expressed genes using a linear model approach along with an empirical Bayes method to better estimate errors in log-fold change.

Partek Genomics Suite was used to perform gene ontology microarray data using GO ANOVA, with analysis restricted to groups with more than two and fewer than 150 genes. The top 100 GO biological function terms were visualized using the REVIGO online software platform (Rudjer Boskovic Institute, Zagreb, Croatia). Gene set enrichment analysis was performed using the Gene Set Enrichment Analysis software platform (Broad Institute, Cambridge, Massachusetts) with publicly-available annotated gene sets (cholesterol_homeostasis, antigen_processing_presentation, fcr_mediated_phagocytosis, phagosome_maturation) obtained from the Molecular Signatures Database (MSigDB). Analysis was run using 10,000 permutations with FDR < 0.25.

### Murine GATA2 Expression Analysis

Use of animals for this study was obtained from the Western University Animal Care Committee under Animal Use Protocol (AUP) 2018-131. Mice were raised under pathogen-free conditions in the West Valley Barrier Facility at Western University. LDLR-deficient mice on a C57NL/6J background (B6.129S7-Ldlrtm1Her/J) were fed either control chow, or high fat chow (17.3%/w protein, 48.7/w carbohydrate, 21.2%/w fat) for 12 weeks. Animals were subsequently sacrificed, with thoracic aortas being subsequently dissected and homogenized. Total RNA was extracted from homogenized tissue and *Gata2* gene expression was assessed *via* real-time qPCR as detailed below.

### THP1 Cell Culture

THP1 human monocytes were cultured in RPMI + 10% FBS and split upon reaching a density of ~2 × 10^6^/ml. To generate THP1 derived macrophages, #1.5 thickness, 18 mm diameter circular coverslips were placed into the wells of a 12-well plate, 1 × 10^5^ THP1 cells placed into each well cultured for 72 h in RPMI + 10% FBS + 100 nM PMA. Cells were then incubated with human oxLDL at a concentration of 100 µg/ml for 72 h (46). oxLDL-treated cells were washed with PBS and plated into fresh, oxLDL-free media prior to use.

### Cloning

GATA2 was sub-cloned from pBabePuro-GATA2 into the pLXV-zsGreen lentiviral vector by restriction cloning, using EcoRI as per the manufacturer’s instructions. GATA2 shRNA was ordered as an oligo (AAGGA TCCAG CAAGG CTCGT TCCTG TTCAT CAAGA GTGAA CAGGA ACGAG CCTTG CTTTT TTACC GGTA A) and cloned into pGFP-C-shLenti by restriction cloning with BamHI and AgeI. These were packaged into a VSV-G pseudotyped lentiviral vector using HEK 293T cells expressing pMD2.G (2 ng, Addgene #12259), pCMV-DR8.2 (5 ng, Addgene #12263), and 2.5 µg of the lentiviral vector. Seventy-two hour post transfection, virus was purified and stored as described previously -80°C (47). THP1 cells were transduced by adding 500 µl of viral isolate to 1 × 10^6^ of THP1 cells with 8 µg/ml polybrene. One week later, transduced cells were fluorescence-activated cell-sorted based on their expression of the zsGreen/GFP marker encoded by the vectors. All primer and shRNA sequences can be found in **Supplemental Table 1**.

### RNAseq

RNA was isolated after THP-1 differentiation with an RNA extraction kit according to manufacturer’s protocol. RNA was stored at -80°C. RNA quantity was measured using a NanoDrop (Thermo Fischer Scientific) and RNA quality was checked using a 2100 Bioanalyzer Instrument (Agilent Technologies Inc., Palo Alto, CA) and RNA 6000 Nani kit (Caliper Life Sciences, Mountain View CA). Samples below an A260/280 of 1.5 and/or RIN of 7 rejected from analysis. RNA samples were processed using Vazyme VAHTS Total RNA-seq Library Prep Kit for Illumina, including rRNA reduction and fragmentation. RNA library size distribution was assessed on an Agilent High Sensitivity DNA Bioanalyzer chip and quantified using the Qubit 2.0 Fluorimeter (Thermo Fischer). The library was sequenced on an Illumina NextSeq 500 as a single end run, 1 x76 bp, using a High Output v2 kit (75 cycles). Fastq data files from BaseSpace were analyzed using Partek Flow (St. Louis, MO) and the sequences were aligned to the Homo sapiens genome using STAR 2.6.1d and annotated using Ensembl v 98. Features with more than 10 reads were normalized by adding 1.0 and using Trimmed Mean of M-values (TMM). Gene Specific Analysis was used to calculate fold change and p-values and identify differentially expressed genes. Resultant filtered gene lists were then analyzed for enriched Gene Ontology and KEGG pathway terms. REVIGO was used to summarize and reduce gene ontology terms, PCA analyses of differentially expressed genes was quantified using Matlab, and KEGG bubble plots were prepared using WebGestalt (48, 49).

### Immunoblotting

At least 5 × 10^5^ cells were lysed in Laemmli’s buffer + 10% β-mercaptoethanol and Halt protease inhibitor cocktail, boiled briefly, separated on a 10% SDS-PAGE gel, and transferred to nitrocellulose membrane. The membrane was blocked for at least 1 h with Tris-buffered saline with 0.1% Tween 20 (TBS-T) + 5% bovine serum albumin in PBS, incubated for 1 h with anti-GATA2 antibody (1:500 dilution) or anti-GAPDH (1:1000) in TBS-T + 2.5% milk powder. The blot was washed 3 × 15 min in TBS-T, and then a 1:10,000 dilution of an appropriate IR700 or IR800 secondary antibody added for 30 min to 1 h in TBS-T + 5% bovine serum albumin in PBS. The blots were washed 3 × 15 min washes in TBS-T and imaged using an Odyssey CLx (LI-COR Biosciences, Lincoln, Nebraska).

### Real-Time PCR

Total RNA was isolated from cells or patient tissues through acid guanidinium thiocyanate-phenol-chloroform extraction using PureZOL RNA isolation reagent. Cells or tissues were suspended in PureZOL, vortexed briefly, and incubated for 5 min at room temperature. Chloroform was added 1:5 v/v and incubated for another 5 min with intermittent agitation. Samples were centrifuged for 15 min at 12,000 ×g and 4°C, then the aqueous layer transferred to a fresh tube to which an equal volume of isopropanol and 0.5 µl of 20 mg/ml glycogen were added. Samples were incubated at -80°C for at least 30 min, centrifuged for 15 min at 12,000 × g and 4°C, and the RNA pellet washed 2× with 75% ethanol, air dried for approximately 5 min at room temperature and reconstituted in a minimal amount of RNase-free ddH_2_O. RNA concentration and quality were measured using a NanoDrop 1000 Spectrophotometer. cDNA was generated using the iScript Select cDNA Synthesis Kit according to the manufacturer’s instructions, with an equal amount of starting RNA an equal mix of random and oligo(dT)20 primers. cDNA concentration and quality were checked using a spectrophotometer prior to use in qPCR reactions. qPCR was performed using the Fast SYBR Green Master Mix and an equal amount of starting cDNA. PCR reactions were run on a QuantStudio 5 Real-Time PCR System for 40 cycles. Relative transcript express was calculated using the ΔΔCt method with 18S used as the housekeeping gene. All RT-PCR primer sequences can be found in **Supplemental Table 1**.

### Phagocytosis and Efferocytosis Assays

Phagocytosis and efferocytosis assays were performed as described previously (43, 50). Briefly, phagocytic targets were generated by washing 10 μl of 5 µm diameter P(S/DVB) polystyrene beads (6,000 × g/1 min) with 1 ml of PBS. Beads were suspended in 100 µl of PBS + 0.1 mg/ml human IgG and incubated at 20°C for 30 min. Beads were then washed as above and suspended in 1 ml of DMEM. Efferocytic targets were prepared by suspending a 4 μmol mixture of 20% phosphatidylserine, 79.9% phosphatidylcholine, and 0.1% biotin-phosphatidylethanolamine in chloroform, and to this adding 10 μl of 3 µm diameter silica beads. After a 1 min vortex, the chloroform was evaporated with nitrogen and the beads suspended in 1 ml of PBS. The beads were then washed 3× as described above and suspended in 1 ml of DMEM. These beads were then added to macrophages at a 10:1 bead:macrophage ratio, spun at 200 × g for 1 min to force contact between the cells and beads, and then incubated for the indicated time. The samples were then fixed with 4% PFA for 20 min, washed, and if required, non-internalized beads detected by incubation for 20 min in PBS + 1:1000 dilution of Cy3-labeled anti-human secondary antibody, or DMEM + 1:500 dilution of Alexa-555 labeled streptavidin. Samples were then washed 3× in PBS or DMEM, mounted on slides with Permafluor, and imaged.

### Lysosome Fusion

Lysosomes in THP1 derived macrophages were loaded with 100 µg/ml 10,000 MW TRITC-conjugated dextran for 16 h, followed by a 90 min chase with serum-free RPMI. IgG-coated beads, prepared as above, were added at a 10:1 bead:macrophage ratio and briefly centrifuged for 1 min at 400 × g, then incubated for 30 min at 37°C and 5% CO_2_. Following incubation, cells were washed 1× with PBS and labelled 1:1,000 with a Cy5-conjugated anti-human IgG secondary antibody to label non-internalized beads. Cells were washed 3 × 5 min with PBS, fixed with 4% PFA for 20 min, washed an additional 3 × 5 min and mounted onto glass slides using Permafluor. Samples were imaged at 63× magnification, using the red (dextran) and far-red (beads) channels.

### Phagosome pH

Human IgG was labeled with pHrodo as per the manufacturer’s instructions, and phagocytic targets prepared as described above using a 500:1 ratio of pHrodo-IgG : Alexa-647-labeled irrelevant antibody. Live cell microscopy was performed at 63× magnification, using point-visiting to image 4–5 locations (20–30 cells) per condition. The media was then replaced with DMEM + phagocytic targets and a 90 min timelapse captured at 2 min/frame using the DIC, red (pHrodo) and far-red filter sets, maintaining the same camera and exposure settings used across all experimental conditions within an individual experiment. At the end of the experiment the media was replaced with high-potassium media + 10 μg/ml nigericin at a pH of 4.0, 5.0, 6.0, and 7.0. The cells were imaged after each media change, thereby providing an *in situ* pH calibration for each phagosome. The resulting images were imported into FIJI, individual beads tracked using the manual tracking plugin and the pHrodo : Alexa-647 ratio measured in each phagosome at each time point and the pH determined using the data from the calibration images. Phagosomal FITC staining was normalized to the integrated FITC intensity across the whole cell at the first time point.

### Oxidant Production

A fluorescence microscopy-based NBT assay protocol was used to assess macrophage NADPH oxidase activity (51). Apoptotic Jurkat cells were generated as per our published protocols (43, 50) and added to PMA-differentiated THP1 cells at a 10:1 Jurkat:macrophage ratio. Samples were centrifuged for 1 min at 400 ×g to force contact between the macrophages and ACs, and NBT was added to the medium to a final concentration of 100 µg/ml. After 1 h incubation at 37°C and 5% CO_2_ cells were washed 3 × 5 min and fixed with 4% PFA for 20 min. Cells were washed 3 × 5 min with PBS and mounted onto slides using Permafluor. Fluorescence of diformazan deposits formed were imaged on using at 63×, using the far-red fluorescence channel.

### Cholesterol Uptake

Cholesterol accumulation was measured by staining with either ORO or Nile Red (NR). ORO solution (1:250 w/v ORO powder in isopropanol) was diluted 3:2 in ddH_2_O and passed through a 0.2 µm filter. Cells were washed 1× with PBS, fixed with 4% PFA for 20 min, washed an additional 3×, washed briefly with 3:2 v/v mixture of isopropanol and ddH_2_O, and allowed to dry. Cells were then covered with the minimum volume of ORO working solution and incubated for 5 min, washed 3 × 5 min with PBS, and mounted for imaging. ORO accumulation was quantified by extracting stained cells with 100% isopropanol for 10 min at room temperature with gentle agitation, and absorbance measured at 518 nm. NR (1 mg/ml in acetone) was diluted 1:100 into PBS or serum-free DMEM. For fixed cell staining, the PBS solution was added to fixed and washed cells for 5 min, followed by 3 × 5 min with PBS. For live-cell staining, the DMEM solution was added to cells in a Leiden chamber, and images acquired every 15 min for 8 h at 63× magnification, using the red fluorescence channel.

### Cholesterol Efflux

Cholesterol efflux was measured as described by Sankaranarayanan et al. (52). Briefly, BODIPY-cholesterol and unlabeled cholesterol (1:5 v/v) were mixed and dried under nitrogen, solubilized in MEM-HEPES (MEM media with 10 mM HEPES, pH 7.4) + 20 mM methyl-β-cyclodextran (MβCD) at a molar ratio of 1:40 cholesterol:MβCD. The mixture sonicated at 37°C for 30 min, placed onto a stirring hot plate pre-heated to 37°C for 3 h. Immediately prior to use this labeling media is sonicated at 37°C for 30 min and filtered through a 0.45 µm filter. Apolipoprotein B (ApoB)-depleted human serum was prepared by adding 1:40 v/v 1M CaCl_2_ to whole human plasma, incubated for 1 h, and serum separated by centrifugation for 5 min at 1,000 × g. ApoB was then precipitated by addition of 20% PEG 8,000 in 200 mM glycine buffer, pH 7.4, at a 2:5 v/v ratio. The mixture was incubated for 20 min and then centrifuged for 30 min at 10,000 × g and 4°C.

THP1 cells were cultured in a 48-well plate to a density of 75,000 cells/well and differentiated into macrophages by culture with 100 nM PMA for 24 h prior. Cells were washed 1× with PBS and incubated with 250 µl/well of labelling media for 1 h, washed 2× with MEM-HEPES, and then incubated with serum-free RPMI + 0.2% BSA for 16 h. Cells were washed 2× with MEM-HEPES, then incubated with MEM-HEPES + 10% apoB-depleted serum for 4 h. All procedures were performed at 37°C and 5% CO_2_. Media from each well was collected, filtered through a 0.45 µm filter and fluorescence intensity (ex. 482 nm, em. 515 nm) was recorded using a Gemini Fluorescence Microplate Reader (Molecular Devices). Negative controls were solubilized with 4% cholic acid for 4 h, the supernatants filtered through a 0.45 µm filter, and fluorescence intensity was recorded and used as the baseline value for total cholesterol present within the cells. The percent cholesterol efflux was calculated as fluorescence intensity of media divided by fluorescence intensity of cholic acid.

### Citrullination

Cells or tissues sections were incubated with wheat germ agglutinin (1:200 in PBS), 10 min at room temperature, then washed three times in PBS and permeabilized for 10 min with a 0.2% Triton X-100 solution at room temperature. Cells were then washed 3 times in PBS, incubated with equal volumes of modification reagent A (0.5% FeCl2, 4.6 M sulfuric acid, and 3.0 M phosphoric acid) and modification reagent B (0.5% 2,3-butanedione monoxime, 0.25% antipyrine, 0.5M acetic acid) at 37°C for 3 h. Cells were then washed three times in PBS, blocked for 1 h with 5% FBS, then incubated for 1 h with anti-modified citrulline or an isotype control at 1 µg/ml in 5% FBS. Cells were washed three times in PBS and incubated with a 1:1000 dilution of Cy3-labeled anti-human secondary antibody in 5% FBS for 1 h. Cells were washed three times with PBS, counter-stained with 2 µg/ml Hoechst for 10 min, washed a final three times and mounted on coverslips with Permafluor mounting medium.

### Statistics and Data Analysis

All datasets were tested for normal distribution using a Shapiro-Wilk test. Normally distributed data is presented as mean ± SEM and analyzed using a Students *T-*test or ANOVA with Tukey correction. Non-parametric data is presented as median ± 95^th^ percentile and analyzed using a Kruskal-Wallis test with Dunn correction. All statistical analyses were performed using Graphpad Prism 6; p values < 0.05 were considered significant.

### Supplemental Materials

Additional figures including sequences for all PCR/RT-PCR primers used in this study, an expanded analysis of patient plaque histology, macrophage CD163 expression, characterization of the GATA2 *in vitro* model, additional RNAseq and microarray analysis, cholesterol efflux analysis, and histology staining controls can be found in the **Supplementary Material**.

## Results

### GATA2 Is Overexpressed in Early-Stage Atherosclerotic Plaque Macrophages

**Figure 1:**
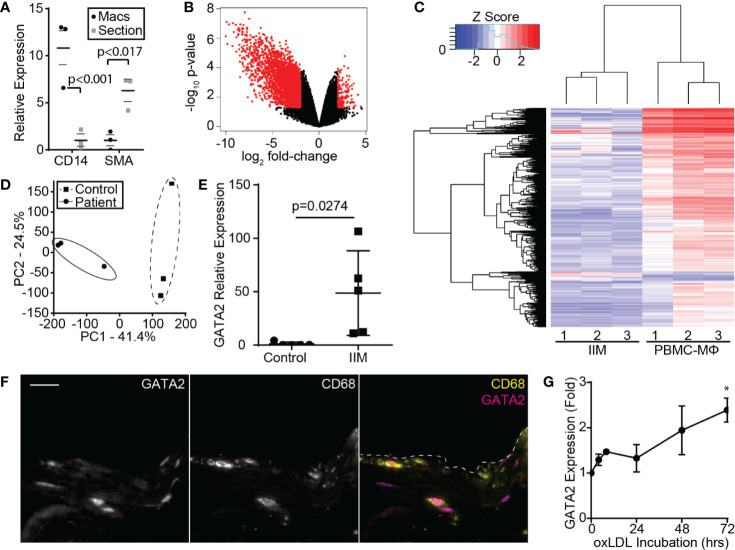
Microarray Analysis of IIMs. **(A)** Enrichment of the macrophage marker CD14 and depletion of the smooth muscle/endothelial marker SMA, following recovery of IIMs by CD163 laser microdissection. Median fold-enrichment ± 95^th^ percentile was calculated following normalization to 16S. **(B)** Volcano plot illustrating the 3,374 genes up- or down-regulated more than 2-fold in IIMs compared to age- and sex-matched monocyte-derived macrophages. **(C)** Microarray heatmap showing the gene expression profile of IIMs to age- and sex-matched monocyte-derived macrophages (PBMC-MΦ). **(D)** Principal component analysis of the gene expression profiles of IIMs versus age- and sex-matched monocyte-derived (Control) macrophages. **(E)** RT-PCR quantification of GATA2 expression in the IIMs versus control macrophages from five additional patients. GATA2 expression is normalized to 16S and then normalized to the mean of the controls. **(F)** Immunofluorescence image of a representative aortic punch biopsy showing the distribution of GATA2 (magenta) and CD68+ macrophages (yellow). Scale bar is 20 µm. **(G)** Change in GATA2 expression in THP1 derived macrophages, measured relative to t = 0, treated with 100 µg/ml oxLDL for 72 h, five independent repeats were performed per time point. Data is quantified as median ± 95% percentile *p < 0.05 (**A**; n = 3), mean ± SEM (**E, G**; n = 5), or is representative of three independent experiments. p-values were calculated using a Mann-Whitney test **(E)** or one-way ANOVA with post-hoc Tukey correction **(G)**.

Human aortic punch samples were obtained from regions of the ascending aorta with minimal visible atherosclerotic disease in patients undergoing coronary artery bypass graft surgery. Approximately 75% of punch samples were found to contain evidence of early stage plaque (stage 1 or 2), as per the histological classification system of Stary (33), and contained significant macrophage infiltrates when stained with anti-CD163 (**Figure S1**). CD163 was used in lieu of the more commonly used macrophage marker CD68, as up to 40% of CD68^+^ cells in plaque are not of macrophage origin (42), while in humans CD163 is a pan-macrophage maker (**Figure S2**) (53). We then isolated macrophages from these human aortic punch biopsy samples using laser capture microdissection using CD163 as a marker (**Figure 1A**). For clarity, these macrophages are henceforth referred to as “Intima Infiltrating Macrophages” (IIMs).

The gene expression profile of these IIMs was then compared to monocyte-derived, M0-differentiated macrophages from age- and sex-matched controls, identifying 3,374 differentially expressed protein-coding genes (**Figures 1B, C****)**. Principal component analysis demonstrated close clustering between patients, indicating that IIMs in early-stage plaques develop a consistent gene expression profile (**Figure 1D**). Gene ontology-based clustering and gene set enrichment analysis were used to identify biological processes that may be perturbed in these IIMs, identifying putative defects in cholesterol homeostasis, the pathways used to engulf and degrade pathogens (phagocytosis) and apoptotic cells (efferocytosis), and antigen processing (**Figure S3**). As expected, given the large number of differentially regulated genes, multiple transcription factors were significantly up- or down-regulated. Surprisingly, pro-inflammatory transcription factors were either not differentially regulated between patients and controls (e.g. NF-κB, Rel, cFos/cJun) or were modestly suppressed in patients (C/EBPβ,γ,ζ, data not shown). Of the transcription factors upregulate in IIMs, only GATA2 had been previously implicated in coronary artery disease in humans (54–56). RT-PCR analysis of IIMs from five additional patients confirmed upregulation of GATA2 in these cells (**Figure 1E**), and immunofluorescence staining determined that GATA2 is predominantly expressed by CD68^+^ cells in these aortic biopsies (**Figure 1F**). We did not see consistent upregulation of GATA2 in a Western-diet fed LDLR^-/-^ mouse model of atherosclerosis (data not shown), nor has it been reported in prior transcriptomics analysis of the LDLR^-/-^ or ApoE^-/-^ atherosclerosis models (57) (GEO Ascension GSE2372). GATA2 expression increased *in vitro* when human monocyte-derived, M0 polarized macrophages were cultured with oxLDL. However, this upregulation was less than 1/10^th^ of that observed in IIMs (**Figure 1G**).

### Characterization of the Macrophage GATA2 Transcriptome

**Figure 2:**
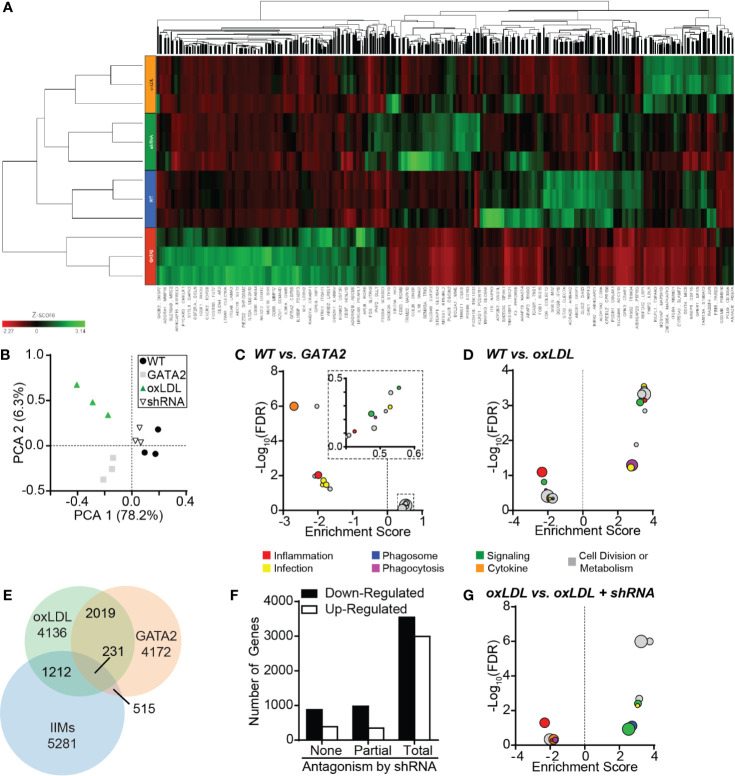
Characterization of the GATA2 Transcriptome. RNAseq analysis was performed in triplicate using THP1-derived macrophages differentiated from wild-type THP1 cells (WT), GATA2-overexpressing THP1 cells (GATA2), WT cells treated for 72 h with 100 µg/ml oxLDL (oxLDL), and oxLDL-treated THP1 cells expressing a GATA2-targeting shRNA (shRNA). **(A)** Heat map of all differentially regulated genes. **(B)** Principal component analysis comparing the gene expression profiles of the four test conditions. **(C, D)** KEGG analysis of the 20 most differentially regulated pathways between wild-type THP1-derived macrophages and either macrophages overexpressing GATA2 **(C)** or treated with oxLDL **(D)**. **(E)** Venn diagram of the overlap in genes dysregulated in THP-1 macrophages expressing a GATA2 transgene or treated with oxLDL versus patient-derived IIMs. **(F)** Number of genes which are up- or downregulated (compared to WT) by oxLDL treatment whose dysregulation is unaffected (None), partially returned to WT-expression (Partial) or completely returned to WT-expression (Total) by a GATA2-targeting shRNA. **(G)** KEGG analysis of the 20 most differentially regulated pathways of oxLDL treated macrophages ± a GATA2-targeting shRNA. n = 3 independent experiments.

Because neither mouse models nor *in vitro* oxLDL stimulation of human monocyte-derived macrophages produced an upregulation of GATA2 to a similar magnitude of that observed in patient IIMs, we prepared a GATA2-overexpressing THP-1 human monocytic cell line (**Figure S4A, B, D**). Both GATA2 overexpression and oxLDL treatment of wild-type cells induced changes to many of the same pathways that were perturbed in IIMs (**Figures 2A, B**), with pathways mediating phagocytosis, phagosome maturation, and immune cell function including those directed against pathogens, being predominantly affected (**Figures 2C, D**, **Figures S5**, **S6**). When the transcriptomics of GATA2 overexpressing and oxLDL treated macrophages were compared, over 50% of differentially expressed genes were found to overlap between them (**Figure 2E**). This co-regulation was concentrated (~80%) in genes that were downregulated in response to oxLDL, suggesting that GATA2 upregulation by oxLDL is largely involved in the suppression of gene expression. GATA2 overexpression *in vitro* also resulted in differential expression of ~14% of the genes which were found to be dysregulated in patient IIMs (**Figure 2E**). The large overlap in genes expression changes induced by GATA2 versus oxLDL led us to explore this phenomenon further by generating a human macrophage cell line expressing a GATA2-suppressing shRNA (**Figures S4C, D**). This shRNA antagonized 86% of the gene expression changes induced by oxLDL, and as expected, was more likely to affect genes which were downregulated by oxLDL (**Figures 2A, F**, **Figure S6**). Pathway analysis demonstrated that the effect of the shRNA was largely to antagonize oxLDL-induced defects in intracellular signaling, inflammatory pathways, and phagosome maturation (**Figure 2G**, **Figure S6**).

### GATA2 Expression Modestly Affects Macrophage Cholesterol Homeostasis and Immunogenicity

Dysregulation of cellular cholesterol processing is a key feature of atherosclerosis, with dysregulation of this process observed in IIMs (**Figure S3B**). RT-PCR analysis of IIMs confirmed that NPC1 and NPC2, which mediate the transfer of endocytosed cholesterol to cytosolic carrier proteins (58), and ABCA1, which mediates the export of cholesterol from cells (6), were downregulated in IIMs (**Figures S7A–C**). RNAseq analysis of our *in vitro* GATA2 model identified genes putatively regulated by GATA2—defined as genes that were up- or downregulated by both oxLDL and GATA2 overexpression, with this change reversed by the GATA2 shRNA. This included genes required for cholesterol import (NPC1), trafficking (OSBPL5), and export (ABCA1 and ABCG1, **Figure S7D**). However, neither GATA2 overexpression nor the GATA2 shRNA had any effect on cholesterol accumulation or export in these cells (**Figures S7E, F**), indicating that GATA2 plays only a minor role in the perturbance of cholesterol homeostasis during atherosclerosis.

**Figure 3:**
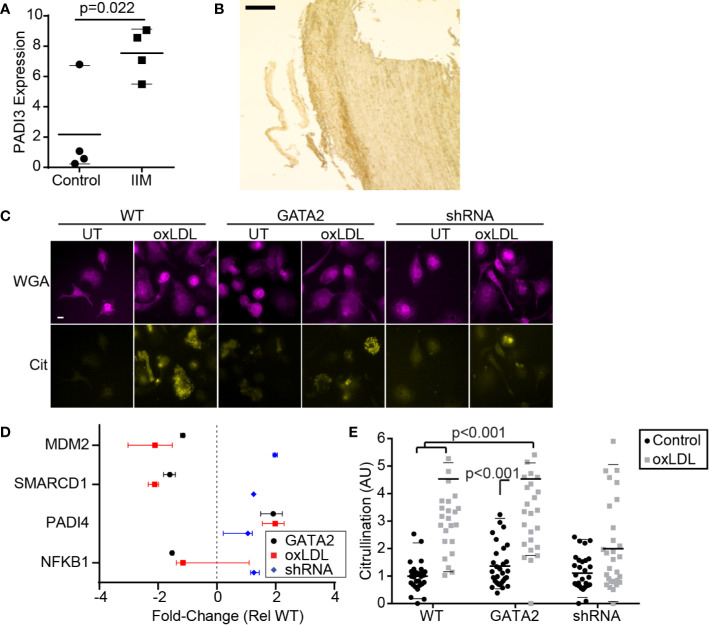
GATA2 Expression is Required for Citrullination. **(A)** RT-PCR quantification of PADI3 expression in IIMs versus age- and sex-matched monocyte-derived macrophage controls (Control). **(B)** Low-magnification image of an aortic punch biopsies stained for citrullinated peptides. Scale bar is 1 mm. **(C)** Anti-citrulline immunofluorescence of THP1-derived macrophages that are either normocholesterolemic (UT) or hypercholesterolemic (oxLDL). Cells are stained with a plasma-membrane marker (WGA) and an anti-citrulline antibody (Cit). Scale bar is 10 μm. **(D)** Changes in gene expression, compared to vector-control, of citrullination-associated genes in THP1-derived macrophages expressing a GATA2 transgene (GATA2), or treated with oxLDL ± a GATA2-targeting shRNA (oxLDL, shRNA). **(E)** Protein citrullination in THP1-derived macrophages expressing a vector-control (WT), GATA2 transgene (GATA2) or GATA2-targeting shRNA (shRNA) in response to normocholesterolemic (Control) or hypercholesterolemic (oxLDL) conditions. Data is representative of **(B, C)**, or quantifies as the median ± 95^th^ percentile **(A, D, E)**, a minimum of three independent experiments. p values were calculated using Students t-test **(A)** or Kruskal-Wallis test with Dunn correction **(D, E)**.

We also observed evidence of atherogenic antigen processing in IIMs such as perturbation of the MHC I and MHC II presentation pathways (**Figure 1C**, **Figure S3E**), as well as upregulation of pathways that generate atherogenic self-antigens. This included proteins involved in citrullination—the deimidation of arginine to form the amino acid citrulline, producing citrullinated self-antigens that contribute to intra-plaque immune complex deposition and are associated with accelerated disease in some patients (59–61). RT-PCR analysis confirmed the upregulation of PADI3 in IIMs (**Figure 3A**), and immunohistochemistry identified citrullinated peptides in the intima of these patients’ biopsies (**Figure 3B**, **Figure S8A**). Similar patterns of citrullination and PADI3 expression were observed in oxLDL-treated macrophages *in vitro* (**Figure 3C**, **Figure S8B**), and RNAseq analysis of our *in vitro* GATA2 model identified several citrullination-associated genes putatively regulated by GATA2 (**Figure 3D**). Using quantitative microscopy, we determined that GATA2 was necessary, but not sufficient, for oxLDL-induced protein citrullination. Specifically, oxLDL was unable to induce citrullination in the absence of GATA2, but GATA2 overexpression alone (e.g. in the absence of oxLDL) was also insufficient to induce citrullination (**Figure 3E**). Together, these data indicate that IIMs are engaged in protein citrullination, with GATA2 playing a synergistic role alongside other oxLDL-induced factors.

### GATA2 Overexpression Impairs Phagocytosis and Efferocytosis

**Figure 4:**
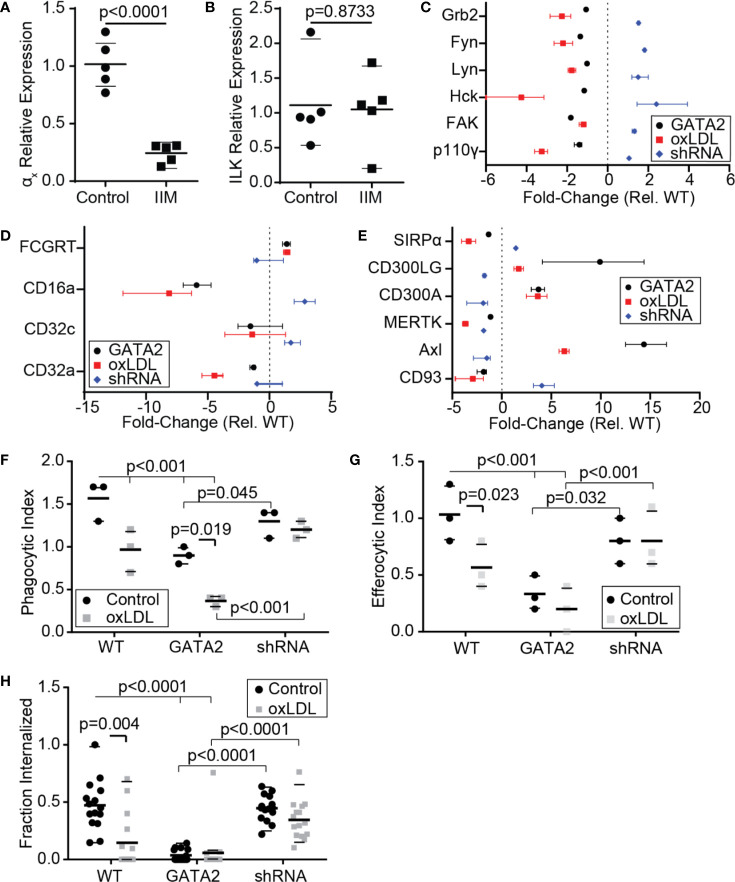
Macrophage Phagocytosis and Efferocytosis are Impaired by GATA2. RT-PCR was used to quantify the expression of α_x_ integrin **(A)** and integrin-linked kinase (ILK, **B**) in IIMs versus age- and sex-matched monocyte-derived macrophages (Control). **(C–E)** Change in gene expression, compared to vector-control, of phagocytosis-associated signaling molecules **(C)**, Fcγ receptors **(D)**, and efferocytic receptors **(E)** in THP1-derived macrophages expressing a GATA2 transgene (GATA2), or treated with oxLDL ± a GATA2-targeting shRNA (oxLDL, shRNA). **(F–H)** Quantification of the effect of normocholesterolemia (Control) or hypercholesterolemia (oxLDL) on Thp1-derived macrophages expressing either a vector control (WT), GATA2 transgene (GATA2), or GATA2-targeting shRNA (shRNA) on the phagocytic uptake of IgG-coated phagocytic mimics **(F)**, the uptake of PtdSer-bearing apoptotic cell mimics **(G)**, or the fraction of apoptotic Jurkat cells efferocytosed over 90 min **(H)**. Data is expressed as individual values and mean ± SEM (A,B), or as median ± 95^th^ percentile **(C–H)**, n = a minimum of three independent experiments, p-values were calculated using Students t-test **(A, B)** or Kruskal-Wallis test with Dunn correction **(C–H)**.

The efferocytic removal of apoptotic cells is a critical atheroprotective mechanism, and failures in this process are prerequisites for the accumulation of uncleared apoptotic cells which form the necrotic core of advanced atherosclerotic plaques (1, 2, 39, 62). Our analysis of IIMs identified a reduction in the expression of multiple genes involved in the efferocytosis of apoptotic cells and the phagocytosis of microbial pathogens (**Figures S3**). RT-PCR confirmed that the α_x_ integrin, a receptor for both complement-opsonized pathogens and CD93-opsonized apoptotic cells, was downregulated in IIMs (**Figure 4A**) (63). Unexpectedly, integrin-linked kinase, which was downregulated in the patients analyzed in our microarray analysis (**Figure 1C**) was not downregulated in the patient cohort used for our RT-PCR assays (**Figure 4B**). Our *in vitro* GATA2 model identified a number of canonical phagocytic/efferocytic signaling molecules, as well as several phagocytic and efferocytic receptors, which were dysregulated in a manner consistent with oxLDL-mediated GATA2 overexpression as the driving factor (**Figures 4C–E**). The phagocytosis of antibody-opsonized particles *in vitro* was profoundly impaired by both culture with oxLDL and GATA2 overexpression, with this defect abrogated by expression of a GATA2 shRNA (**Figure 4F**). The same trend was observed in efferocytosis, with both the total number of apoptotic mimics engulfed per macrophage (**Figure 4G**) and the efficacy of apoptotic cell uptake (**Figure 4H**) impaired by culture with oxLDL or GATA2 overexpression, and with expression of a GATA2 shRNA restoring normal efferocytic capacity. Combined, these data indicate that GATA2 overexpression impairs the ability of macrophages to recognize and engulf both phagocytic and efferocytic targets.

### GATA2 Overexpression Impairs Phagosome/Efferosome Maturation

**Figure 5:**
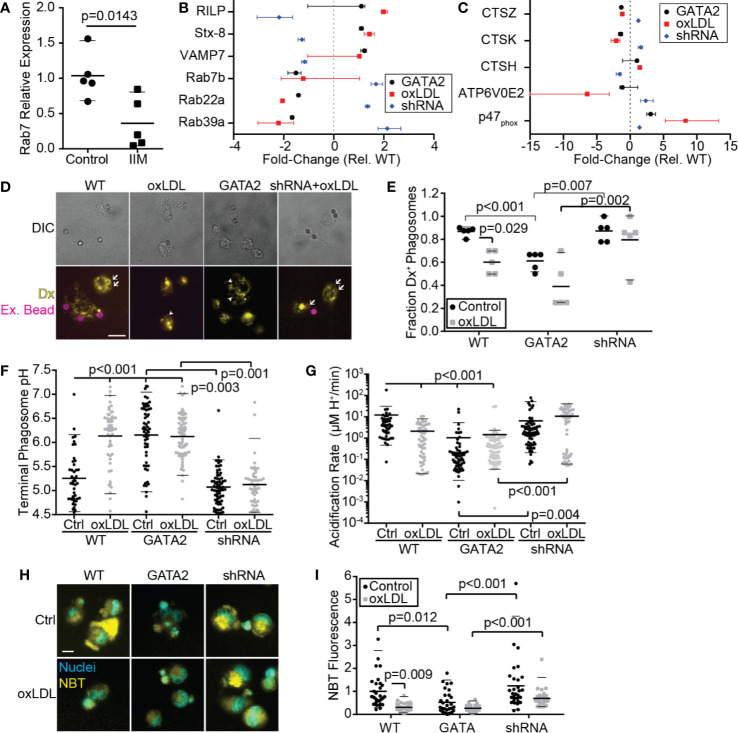
Phagosome/Efferosome Maturation is Impaired by GATA2. **(A)** RT-PCR quantification of Rab7 expression in IIMs versus age- and sex-matched monocyte-derived macrophages (Control). **(B, C)** Change in gene expression in THP1-derived macrophages, compared to vector-control, of genes required for phagosome trafficking **(B)** or degradation of phagosome contents **(C)**. **(D, E)** Representative images **(D)** and quantification **(E)** of the effect normocholesterolemia (Control) or hypercholesterolemia (oxLDL) on THP1-derived macrophages expressing either a vector-control (WT), GATA2 transgene (GATA2) or GATA2-targeting shRNA (shRNA) on the fusion of dextran-loaded lysosomes (Yellow, Dx) with IgG-coated phagocytic targets. Dextran-positive targets are indicated with arrows; dextran-negative internalized targets are indicated with arrowheads. Non-internalized targets are stained with streptavidin-647 (Magenta, Ex. Beads). Fusion is quantified 30 min after phagocytosis. **(F, G)** Terminal phagosome pH **(F)** and phagosome acidification rate during the first 5 min following phagosome closure **(G)**, as measured using pHrhodo ratiometric imaging of THP1-derived macrophages. **(H, I)** Representative images **(H)** and quantitation **(I)** of superoxide production 90 min after efferocytosis of apoptotic Jurkat cells by THP1-derived macrophages, as measured by NBT fluorescence. Data are expressed as mean ± SEM **(A)**, as median ± 95^th^ percentile **(B, C, E–G, I)**, or is representative **(D, H)** of a minimum of 3 independent experiments. p values were calculated using Students t-test **(A)** or Kruskal-Wallis test with Dunn correction **(C–G, E)**. Scale bars are 10 µm **(D)** or 20 µm **(H)**.

In addition to downregulation of receptors and signaling molecules associated with the phagocytic and efferocytic uptake of particulates, IIMs also downregulated multiple genes required for the processing of microbes and apoptotic cells following engulfment (**Figure 1C**, **Figure S3**). This included downregulation of Rab7, which is required for phagosome/efferosome fusion with lysosomes (64), multiple subunits of the vacuolar ATPase which acidifies maturing phagosomes/efferosomes (65), and two subunits of the NADPH oxidase complex which produces reactive oxygen species to assist in pathogen/apoptotic cell degradation (66). RT-PCR analysis confirmed the downregulation of Rab7 in IIMs (**Figure 5A**). RNAseq analysis of our *in vitro* GATA2 model identified multiple intracellular trafficking regulators and lysosomal proteins involved in proteolysis, acidification and reactive oxygen species production, whose expression was perturbed in a GATA2-dependent fashion (**Figures 5B, C**). Consistent with these observations, phagosome-lysosome fusion defects were observed in oxLDL-treated and GATA2-overexpressing cells, with this impairment prevented by expression of a GATA2 shRNA (**Figures 5D, E**). In addition to lysosome fusion defects, oxLDL also induced a GATA2-dependent suppression of terminal phagosome pH (**Figure 5F**). This was not merely a result of poor lysosome-phagosome fusion, as the acidification rate of phagosomes in the first 5 min [i.e. prior to lysosome fusion (44)] was also impaired by oxLDL in a GATA2-dependent manner (**Figure 5G**). Using the oxidant-sensitive dye nitro blue tetrazolium (NBT), we next determined that, as with lysosome fusion and phagosome acidification, the production of superoxide was impaired by oxLDL in a GATA2-dependent manner (**Figures 5H, I**). Combined, these data demonstrate that GATA2 overexpression in macrophages causes impairments in multiple processes required for the efficient degradation of apoptotic cells and pathogens taken up *via* phagocytosis and efferocytosis.

## Discussion

In this study we identify a previously unreported macrophage polarization state present in the early stages of human atherosclerotic plaque formation. This polarization state differs from other previously reported atherosclerosis-associated polarization states, most of which have been identified in advanced plaque or in animal models of disease. The polarization state we have identified is characterized by downregulation of atheroprotective processes including cholesterol homeostasis and efferocytosis but lacks the upregulation of inflammatory genes observed in macrophages from more advanced stages of disease. This polarization state was driven, in part, by the upregulation of the transcription factor GATA2, which *in vitro* impaired both the efferocytic uptake of apoptotic cells and their subsequent degradation. Our data implicates GATA2 as a driver of macrophage dysfunction in early human atherosclerosis and provides a detailed map of the transcriptional changes occurring in macrophages during the early stages of plaque formation.

Macrophages are phenotypically plastic cells with flexible transcriptional programs driven by tissue-specific cues, systemic inflammatory signals, age-associated transcriptional changes, and the developmental history of the macrophage (24, 67). These polarization states are also highly plastic and can rapidly respond to changes in the local microenvironment. For example, M1 and M2 macrophages undergo rapid repolarization *via* a Nrf2-driven transcriptional program into an atherogenic polarization state upon exposure to modified LDL (moLDL) (28). In this study, we determined that macrophages isolated from early-stage human atherosclerotic plaque lack upregulation of classical M1 (CD80/86, iNOS) and M2 (Arg1, CD206) markers, as well as markers previously reported in macrophages from late-stage plaque (TREM2, Nrf2, Atf1) (27, 28, 32). Instead, these cells downregulated many genes involved in the processes of efferocytosis, cholesterol processing, and the degradative pathway required for the removal of apoptotic cells and moLDL, while upregulating immunogenic enzymes such as PADI3. This expression pattern is consistent with the events proposed to occur in early-stage plaque, in which the accumulation of lipid-laden macrophages is among the first identifiable histological features (33). This novel macrophage polarization state appears to be the result of a GATA2-driven transcriptional program. Indeed, our RNAseq data show that overexpression of GATA2 in a human macrophage cell line *in vitro* is sufficient to recapitulate perturbations in pathways involved in efferocytosis/phagocytosis and immune cell function, and antagonizing GATA2 upregulation reverses many of these perturbations. Taken together, these data indicate that GATA2 upregulation in IIMs drive decreased expression of many genes required for the uptake and processing of apoptotic cells, setting the stage for the accumulation of necrotic foam cells which typifies later stages of plaque development (38, 62, 68, 69).

Previous genetic linkage studies have shown that single nucleotide polymorphisms (SNPs) in GATA2 are associated with coronary artery disease and diabetes (54). GATA2 functions as both a transcriptional activator and repressor under different contexts (70) and has been best studied as a mediator of the early stages of myelopoiesis, where it must be downregulated for completion of monocyte development (55, 56, 71). The importance of GATA2 in myelopoiesis is illustrated by patients with inactivating mutations in GATA2, many of whom develop MonoMAC syndrome, a primary immunodeficiency characterized by a near absence of circulating myeloid, NK and B cells (72, 73). While the role of GATA2 in hematopoiesis is well established, very little is known of its role in mature myeloid cells. GATA2 expression in peripheral macrophages has been reported in rat alveolar macrophages responding to the fungal pathogen *Pneumocystis carinii*, where inhibition of GATA2 expression enhanced *P. carinii*’s suppressive effect on phagocytosis (74, 75). GATA2 is also expressed at low levels in myeloid dendritic cells, where its role remains undefined (76). Our data demonstrates that GATA2 is upregulated in response to oxLDL and appears to mediate several well-known atherogenic changes in macrophages, most significantly, reduced efferocytic capacity and impaired efferosome maturation. Interestingly, we also observed that GATA2 overexpression led to increased cholesterol efflux from macrophages *in vitro*. Taken together, these data suggest that GATA2 may initially be upregulated in response to the cellular stress caused by cholesterol accumulation, but its upregulation is ultimately maladaptive through disrupting other atheroprotective functions. We did not observe significant expression of pro-inflammatory genes in patient-derived IIMs, nor did GATA2 over-expression generate an inflammatory gene expression profile. This was unexpected, as in mice GATA2 expression is known to enhance inflammatory mediator production by adipocytes, and in mouse macrophages, GATA2 is upregulated by TLR4 signaling after which GATA2 synergizes with MAPK signaling to induce IL-1β transcription (77, 78). One possible explanation for this discrepancy is that humans and mice rely on different promotors for the induction of GATA2 transcription in hematopoietic cells, with human GATA2 transcripts preferentially transcribed from the distal (IS) exon and promotor, whereas mice preferentially utilize the proximal (IG) exon and promotor (79, 80). This would place GATA2 transcription under the control of different promotors, potentially reducing the inflammation-induced expression of GATA2 in humans, relative to mice. Consistent with this hypothesis, we observed a smaller increase in GATA2 expression in human macrophages treated with the TLR4 ligand oxLDL than has been reported in mouse macrophages treated with the TLR4 ligand LPS (14, 78).

Due to its role in hematopoiesis, it is tempting to hypothesize that GATA2 expression may revert IIMs to an earlier developmental state. However, the transcriptional profile we have identified in these cells correlates poorly with the reported transcriptome of developing myeloid cells (81). Alternatively, GATA2 may be upregulated in order to induce the proliferation of macrophages within the plaque. GATA2 is an established positive regulator of cell cycle, and macrophage proliferation is required for plaque development (35, 55). Consistent with this possibility, our GO analysis of GATA2 over-expressing cells identified a modest enrichment in the upregulation of genes associated with cell cycle. Notably, GATA2 upregulation is not observed during later stages of human disease (27, 30), suggesting that other processes act to suppress efferocytosis as disease progresses. Indeed, it is well established that inflammatory (M1-polarized) macrophages tend to be poorly efferocytic, in large part due to downregulation of efferocytic receptors, and furthermore, that the inflammatory conditions in the plaque induce the cleavage of efferocytic receptors such as MERTK and increase the expression of “don’t eat me” signals such as CD47 (39, 44, 82, 83). Thus, it appears that GATA2 impairs efferocytosis during the early stages of plaque development when minimal inflammation is present in the forming plaque, with this suppression of efferocytosis later supplanted by inflammatory macrophage differentiation.

In this study, we examined gene expression changes in patient macrophages by microarray using macrophages differentiated *in vitro* from peripheral blood monocytes as a control. The lack of a more comparable source of control macrophages is a significant drawback of this approach, yet a suitable source of control cells is difficult to identify. While there is a population of macrophages in the healthy artery, these are primarily located within the adventitia (22, 84) and are of embryonic origin, whereas plaque-resident macrophages are predominantly hematopoietic (22, 85, 86). Thus, the use of monocyte-derived macrophages from healthy age- and sex-matched controls recapitulates the correct ontology and age but lacks the tissue-specific cues encountered in the vascular intima, and our results must be interpreted accordingly. Another limitation of our approach is that while the plaques we identified in our aortic punch samples appear to be in the early stage of development from a histological perspective, they are isolated from patients with advanced disease in the coronary circulation. Ergo, some of the observed gene expression changes may be due to systemic effects of atherosclerosis, rather than changes specific to early-stage plaque development. Undoubtedly, some of the difference in gene expression observed in patient IIMs is due to the nature of our controls, however, many of the features that we identify as unique in this macrophage population are consistent with our expectations of macrophage dysfunction during early stages of atherosclerosis, and are not obviously connected to inherent differences between tissue versus cultured macrophages (34–37, 87, 88). These features include marked downregulation of genes involved in cholesterol homeostasis, phagocytosis, efferocytosis, phagosome/efferosome maturation, and antigen processing and presentation. Nevertheless, further studies examining differences in gene expression between circulating monocytes and plaque-resident macrophages will help elucidate the specific transcriptomic alterations associated with the differentiation of monocytes to macrophages within the atherosclerotic plaque.

In summary, we are the first to report that GATA2 is upregulated in macrophages in early-stage human atherosclerotic plaque and that GATA2 overexpression induces efferocytic defects that are known to contribute to atherogenesis. Significantly, GATA2 upregulation appears to mediate a sizeable portion of the transcriptomic changes in macrophages induced by atherogenic factors such as oxLDL. This GATA2-mediated dysregulation of macrophage activity appears to be part of an aberrant polarization state which arises early in plaque development. As such, targeting of GATA2 and the processes downstream of GATA2 activation may represent a novel therapeutic opportunity to limit plaque development by targeting defective efferocytosis within the atherosclerotic plaque.

## Data Availability Statement

The datasets presented in this study can be found in online repositories. The names of the repository/repositories and accession number(s) can be found below: https://dataverse.scholarsportal.info/dataverse/Heit-GATA2/, https://doi.org/10.5683/SP2/WYSAA0.

## Ethics Statement

The studies involving human participants were reviewed and approved by Office of Human Research Ethics at Western University Health Sciences Research Ethics Board. An exception to the requirement of obtaining written informed consent was granted by the Research Ethics Board as this was a discarded tissue study. The animal study was reviewed and approved by Western University Animal Care Committee University of Western Ontario.

## Author Contributions

Unless otherwise stated, CY performed all experiments, with assistance from JH and NS, and authored the manuscript. AV and MR performed the RNAseq analysis. JA and LB performed the citrullination staining and provided mouse aortic samples. EP, RJ, and JD created and packaged the lentiviral vectors. AN performed all surgeries. BH oversaw the study and aided in the authoring of the manuscript with CY. All authors contributed to the article and approved the submitted version.

## Funding

This study was funded by a Heart and Stroke Foundation of Canada Grant-In-Aid and an Ontario Ministry of Research and Innovation Early Research Award to BH. CY was funded by a Vanier PhD Scholarship and Canadian Institutes for Health Research (CIHR) MD/PhD studentship. JD was funded by a CIHR Operating Grant MOP-389413. EP was funded by an Alexander Graham Bell Doctoral Canada Graduate Scholarship from the Natural Sciences and Engineering Council. JA was funded by the Bone and Joint Institute at the University of Western Ontario. LB was funded by the Arthritis Society Strategic Operating Grant (YIO-14-123) and the Program of Experimental Medicine at the University of Western Ontario. The funding agencies had no role in study design, data collection and analysis, decision to publish, or preparation of the manuscript.

## Conflict of Interest

The authors declare that the research was conducted in the absence of any commercial or financial relationships that could be construed as a potential conflict of interest.

## Abbreviations

ApoB, apolipoprotein B; FBS, Fetal Bovine Serum; IIM, intima-infiltrating macrophage; MβCD, methyl-β-cyclodextran; moLDL, modified LDL; NBT, nitro blue tetrazolium; NR, Nile Red; ORO, Oil-Red-O; oxLDL, oxidized Low-Density Lipoprotein; PBS, Phosphate Buffered Saline; PFA, Paraformaldehyde; SFK, Src-family kinase; TBS-T, Tris-buffered saline with 0.1% Tween 20; TLR, Toll-like receptor.

## References

[1] Thorp E Cui D Schrijvers DM Kuriakose G Tabas I Mertk receptor mutation reduces efferocytosis efficiency and promotes apoptotic cell accumulation and plaque necrosis in atherosclerotic lesions of apoe-/- mice. Arterioscler Thromb Vasc Biol (2008) 28:1421–8. 10.1161/ATVBAHA.108.167197 PMC2575060 18451332

[2] Foks AC Engelbertsen D Kuperwaser F Alberts-Grill N Gonen A Witztum JL Blockade of Tim-1 and Tim-4 Enhances Atherosclerosis in Low-Density Lipoprotein Receptor-Deficient Mice. Arterioscler Thromb Vasc Biol (2016) 36:456–65. 10.1161/ATVBAHA.115.306860 PMC4853762 26821944

[3] Wan E Yeap X-YY Dehn S Terry RL Novak ML Zhang S Enhanced Efferocytosis of Apoptotic Cardiomyocytes Through MER Tyrosine Kinase Links Acute Inflammation Resolution to Cardiac Repair After Infarction. Circ Res (2013) 113:1004–12. 10.1161/CIRCRESAHA.113.301198 PMC3840464 23836795

[4] Elliott MR Koster KM Murphy PS Efferocytosis Signaling in the Regulation of Macrophage Inflammatory Responses. J Immunol (2017) 198:1387–94. 10.4049/jimmunol.1601520 PMC5301545 28167649

[5] Werb Z Cohn ZA Cholesterol metabolism in the macrophage. I. The regulation of cholesterol exchange. J Exp Med (1971) 134:1545–69. 10.1084/jem.134.6.1545 PMC2139099 5126640

[6] Chistiakov DA Bobryshev YV Orekhov AN Macrophage-mediated cholesterol handling in atherosclerosis. J Cell Mol Med (2016) 20:17–28. 10.1111/jcmm.12689 26493158 PMC4717859

[7] Kellner-Weibel G Jerome WG Small DM Warner GJ Stoltenborg JK Kearney MA Effects of intracellular free cholesterol accumulation on macrophage viability: a model for foam cell death. Arterioscler Thromb Vasc Biol (1998) 18:423–31. 10.1161/01.atv.18.3.423 9514411

[8] Goo Y-H Yechoor VK Paul A Transcriptional profiling of foam cells in response to hypercholesterolemia. Genomics Data (2016) 9:37–9. 10.1016/j.gdata.2016.06.006 PMC4925893 27408807

[9] Brown AJ Mander EL Gelissen IC Kritharides L Dean RT Jessup W Cholesterol and oxysterol metabolism and subcellular distribution in macrophage foam cells. Accumulation of oxidized esters in lysosomes. J Lipid Res (2000) 41:226–37. 10681406

[10] Tangirala RK Jerome WG Jones NL Small DM Johnson WJ Glick JM Formation of cholesterol monohydrate crystals in macrophage-derived foam cells. J Lipid Res (1994) 35:93–104. 8138726

[11] Feng B Yao PM Li Y Devlin CM Zhang D Harding HP The endoplasmic reticulum is the site of cholesterol-induced cytotoxicity in macrophages. Nat Cell Biol (2003) 5:781–92. 10.1038/ncb1035 12907943

[12] van Tits LJH Stienstra R van Lent PL Netea MG Joosten LAB Stalenhoef AFH Oxidized LDL enhances pro-inflammatory responses of alternatively activated M2 macrophages: a crucial role for Krüppel-like factor 2. Atherosclerosis (2011) 214:345–9. 10.1016/j.atherosclerosis.2010.11.018 21167486

[13] Stewart CR Stuart LM Wilkinson K van Gils JM Deng J Halle A CD36 ligands promote sterile inflammation through assembly of a Toll-like receptor 4 and 6 heterodimer. Nat Immunol (2010) 11:155–61. 10.1038/ni.1836 PMC2809046 20037584

[14] Chávez-Sánchez L Garza-Reyes MG Espinosa-Luna JE Chávez-Rueda K Legorreta-Haquet MV Blanco-Favela F The role of TLR2, TLR4 and CD36 in macrophage activation and foam cell formation in response to oxLDL in humans. Hum Immunol (2014) 75:322–9. 10.1016/j.humimm.2014.01.012 24486576

[15] Tabas I Consequences and therapeutic implications of macrophage apoptosis in atherosclerosis: the importance of lesion stage and phagocytic efficiency. Arterioscler Thromb Vasc Biol (2005) 25:2255–64. 10.1161/01.ATV.0000184783.04864.9f 16141399

[16] Schrijvers DM De Meyer GRY Kockx MM Herman AG Martinet W Phagocytosis of apoptotic cells by macrophages is impaired in atherosclerosis. Arterioscler Thromb Vasc Biol (2005) 25:1256–61. 10.1161/01.ATV.0000166517.18801.a7 15831805

[17] Tabas I Apoptosis and plaque destabilization in atherosclerosis: the role of macrophage apoptosis induced by cholesterol. Cell Death Differ (2004) 11 Suppl 1:S12–16. 10.1038/sj.cdd.4401444 15143347

[18] McNeill E Iqbal AJ Jones D Patel J Coutinho P Taylor L Tracking Monocyte Recruitment and Macrophage Accumulation in Atherosclerotic Plaque Progression Using a Novel hCD68GFP/ApoE-/- Reporter Mouse-Brief Report. Arterioscler Thromb Vasc Biol (2017) 37:258–63. 10.1161/ATVBAHA.116.308367 PMC5274540 27908893

[19] Badrnya S Schrottmaier WC Kral JB Yaiw K-C Volf I Schabbauer G Platelets mediate oxidized low-density lipoprotein-induced monocyte extravasation and foam cell formation. Arterioscler Thromb Vasc Biol (2014) 34:571–80. 10.1161/ATVBAHA.113.302919 24371083

[20] Lopes-Virella MF Virella G Pathogenic role of modified LDL antibodies and immune complexes in atherosclerosis. J Atheroscler Thromb (2013) 20:743–54. 10.5551/jat.19281 23965492

[21] Lichtman AH Clinton SK Iiyama K Connelly PW Libby P Cybulsky MI Hyperlipidemia and atherosclerotic lesion development in LDL receptor-deficient mice fed defined semipurified diets with and without cholate. Arterioscler Thromb Vasc Biol (1999) 19:1938–44. 10.1161/01.ATV.19.8.1938 10446074

[22] Ensan S Li A Besla R Degousee N Cosme J Roufaiel M Self-renewing resident arterial macrophages arise from embryonic CX3CR1(+) precursors and circulating monocytes immediately after birth. Nat Immunol (2016) 17:159–68. 10.1038/ni.3343 26642357

[23] Lavine KJ Epelman S Uchida K Weber KJ Nichols CG Schilling JD Distinct macrophage lineages contribute to disparate patterns of cardiac recovery and remodeling in the neonatal and adult heart. Proc Natl Acad Sci U.S.A. (2014) 111:16029–34. 10.1073/pnas.1406508111 PMC4234568 25349429

[24] Hoeksema MA Glass CK Nature and nurture of tissue-specific macrophage phenotypes. Atherosclerosis (2019) 281:159–67. 10.1016/j.atherosclerosis.2018.10.005 PMC6399046 30343819

[25] Mahbub S Deburghgraeve CR Kovacs EJ Advanced age impairs macrophage polarization. J Interferon Cytokine Res (2012) 32:18–26. 10.1089/jir.2011.0058 22175541 PMC3255514

[26] Davis MJ Tsang TM Qiu Y Dayrit JK Freij JB Huffnagle GB Macrophage M1/M2 polarization dynamically adapts to changes in cytokine microenvironments in Cryptococcus neoformans infection. MBio (2013) 4:e00264–13. 10.1128/mBio.00264-13 PMC3684832 23781069

[27] Cochain C Vafadarnejad E Arampatzi P Pelisek J Winkels H Ley K Single-Cell RNA-Seq Reveals the Transcriptional Landscape and Heterogeneity of Aortic Macrophages in Murine Atherosclerosis. Circ Res (2018) 122:1661–74. 10.1161/CIRCRESAHA.117.312509 29545365

[28] Kadl A Meher AK Sharma PR Lee MY Doran AC Johnstone SR Identification of a novel macrophage phenotype that develops in response to atherogenic phospholipids via Nrf2. Circ Res (2010) 107:737–46. 10.1161/CIRCRESAHA.109.215715 PMC2941538 20651288

[29] Sussan TE Jun J Thimmulappa R Bedja D Antero M Gabrielson KL Disruption of Nrf2, a key inducer of antioxidant defenses, attenuates ApoE-mediated atherosclerosis in mice. PloS One (2008) 3:e3791. 10.1371/journal.pone.0003791 19023427 PMC2582492

[30] Stöger JL Gijbels MJJ van der Velden S Manca M van der Loos CM Biessen EAL Distribution of macrophage polarization markers in human atherosclerosis. Atherosclerosis (2012) 225:461–8. 10.1016/j.atherosclerosis.2012.09.013 23078881

[31] Boyle JJ Harrington HA Piper E Elderfield K Stark J Landis RC Coronary intraplaque hemorrhage evokes a novel atheroprotective macrophage phenotype. Am J Pathol (2009) 174:1097–108. 10.2353/ajpath.2009.080431 PMC2665768 19234137

[32] Boyle JJ Johns M Kampfer T Nguyen AT Game L Schaer DJ Activating transcription factor 1 directs Mhem atheroprotective macrophages through coordinated iron handling and foam cell protection. Circ Res (2012) 110:20–33. 10.1161/CIRCRESAHA.111.247577 22052915

[33] Stary HC Chandler AB Dinsmore RE Fuster V Glagov S Insull W A definition of advanced types of atherosclerotic lesions and a histological classification of atherosclerosis. A report from the Committee on Vascular Lesions of the Council on Arteriosclerosis, American Heart Association. Circulation (1995) 92:1355–74. 10.1161/01.CIR.92.5.1355 7648691

[34] Swirski FK Libby P Aikawa E Alcaide P Luscinskas FW Weissleder R Ly-6Chi monocytes dominate hypercholesterolemia-associated monocytosis and give rise to macrophages in atheromata. J Clin Invest (2007) 117:195–205. 10.1172/JCI29950 17200719 PMC1716211

[35] Tang J Lobatto ME Hassing L van der Staay S van Rijs SM Calcagno C Inhibiting macrophage proliferation suppresses atherosclerotic plaque inflammation. Sci Adv (2015) 1:e1400223. 10.1126/sciadv.1400223 26295063 PMC4539616

[36] Koller D Hackl H Bogner-Strauß JG Hermetter A Effects of oxidized phospholipids on gene expression in RAW 264.7 macrophages: a microarray study. PloS One (2014) 9:e110486. 10.1371/journal.pone.0110486 25333283 PMC4204898

[37] Ho M-M Fraser DA Transcriptome data and gene ontology analysis in human macrophages ingesting modified lipoproteins in the presence or absence of complement protein C1q. Data Br (2016) 9:362–7. 10.1016/j.dib.2016.09.008 PMC5035341 27699187

[38] Linton MF Babaev VR Huang J Linton EF Tao H Yancey PG Macrophage Apoptosis and Efferocytosis in the Pathogenesis of Atherosclerosis. Circ J (2016) 80:2259–68. 10.1253/circj.CJ-16-0924 PMC5459487 27725526

[39] Cai B Thorp EB Doran AC Sansbury BE Daemen MJAP Dorweiler B MerTK receptor cleavage promotes plaque necrosis and defective resolution in atherosclerosis. J Clin Invest (2017) 127:1–5. 10.1172/JCI90520 28067670 PMC5272169

[40] Tong Q Dalgin G Xu H Ting CN Leiden JM Hotamisligil GS Function of GATA transcription factors in preadipocyte-adipocyte transition. Science (2000) 290:134–8. 10.1126/science.290.5489.134 11021798

[41] Schindelin J Arganda-Carreras I Frise E Kaynig V Longair M Pietzsch T Fiji: an open-source platform for biological-image analysis. Nat Methods (2012) 9:676–82. 10.1038/nmeth.2019 PMC3855844 22743772

[42] Albarrán-Juárez J Kaur H Grimm M Offermanns S Wettschureck N Lineage tracing of cells involved in atherosclerosis. Atherosclerosis (2016) 251:445–53. 10.1016/j.atherosclerosis.2016.06.012 27320174

[43] Evans AL Blackburn JWD Yin C Heit B Quantitative Efferocytosis Assays. Methods Mol Biol (2017) 1519:25–41. 10.1007/978-1-4939-6581-6_3 27815871

[44] Yin C Kim Y Argintaru D Heit B Rab17 mediates differential antigen sorting following efferocytosis and phagocytosis. Cell Death Dis (2016) 7:e2529. 10.1038/cddis.2016.431 28005073 PMC5261003

[45] Gentleman RC Carey VJ Bates DM Bolstad B Dettling M Dudoit S Bioconductor: open software development for computational biology and bioinformatics. Genome Biol (2004) 5:R80. 10.1186/gb-2004-5-10-r80 15461798 PMC545600

[46] Lara-Guzmán OJ Gil-Izquierdo Á Medina S Osorio E Álvarez-Quintero R Zuluaga N Oxidized LDL triggers changes in oxidative stress and inflammatory biomarkers in human macrophages. Redox Biol (2018) 15:1–11. 10.1016/j.redox.2017.11.017 29195136 PMC5723280

[47] Pawlak EN Dirk BS Jacob RA Johnson AL Dikeakos JD The HIV-1 accessory proteins Nef and Vpu downregulate total and cell surface CD28 in CD4+ T cells. Retrovirology (2018) 15:6. 10.1186/s12977-018-0388-3 29329537 PMC5767034

[48] Supek F Bošnjak M Škunca N Šmuc T REVIGO summarizes and visualizes long lists of gene ontology terms. PloS One (2011) 6:e21800. 10.1371/journal.pone.0021800 21789182 PMC3138752

[49] Liao Y Wang J Jaehnig EJ Shi Z Zhang B WebGestalt 2019: gene set analysis toolkit with revamped UIs and APIs. Nucleic Acids Res (2019) 47:W199–205. 10.1093/nar/gkz401 PMC6602449 31114916

[50] Taruc K Yin C Wootton DG Heit B Quantification of Efferocytosis by Single-cell Fluorescence Microscopy. J Vis Exp (2018) 18:58149. 10.3791/58149 PMC6128118 30176011

[51] Trinh LA McCutchen MD Bonner-Fraser M Fraser SE Bumm LA McCauley DW Fluorescent in situ hybridization employing the conventional NBT/BCIP chromogenic stain. Biotechniques (2007) 42:756–9. 10.2144/000112476 17612300

[52] Sankaranarayanan S Kellner-Weibel G de la Llera-Moya M Phillips MC Asztalos BF Bittman R A sensitive assay for ABCA1-mediated cholesterol efflux using BODIPY-cholesterol. J Lipid Res (2011) 52:2332–40. 10.1194/jlr.D018051 PMC3220299 21957199

[53] Nguyen TT Schwartz EJ West RB Warnke RA Arber DA Natkunam Y Expression of CD163 (hemoglobin scavenger receptor) in normal tissues, lymphomas, carcinomas, and sarcomas is largely restricted to the monocyte/macrophage lineage. Am J Surg Pathol (2005) 29:617–24. 10.1097/01.pas.0000157940.80538.ec 15832085

[54] Muiya NP Wakil S Al-Najai M Tahir AI Baz B Andres E A study of the role of GATA2 gene polymorphism in coronary artery disease risk traits. Gene (2014) 544:152–8. 10.1016/j.gene.2014.04.064 24786211

[55] Nandakumar SK Johnson K Throm SL Pestina TI Neale G Persons DA Low-level GATA2 overexpression promotes myeloid progenitor self-renewal and blocks lymphoid differentiation in mice. Exp Hematol (2015) 43:565–77.e1–10. 10.1016/j.exphem.2015.04.002 25907033

[56] Collin M Dickinson R Bigley V Haematopoietic and immune defects associated with GATA2 mutation. Br J Haematol (2015) 169:173–87. 10.1111/bjh.13317 PMC4409096 25707267

[57] Yang X Peterson L Thieringer R Deignan JL Wang X Zhu J Identification and validation of genes affecting aortic lesions in mice. J Clin Invest (2010) 120:2414–22. 10.1172/JCI42742 PMC2898611 20577049

[58] Luo J Jiang L Yang H Song B-L Routes and mechanisms of post-endosomal cholesterol trafficking: A story that never ends. Traffic (2017) 18:209–17. 10.1111/tra.12471 28191915

[59] Geraldino-Pardilla L Giles JT Sokolove J Zartoshti A Robinson WH Budoff M Association of Anti-Citrullinated Peptide Antibodies With Coronary Artery Calcification in Rheumatoid Arthritis. Arthritis Care Res (Hoboken) (2017) 69:1276–81. 10.1002/acr.23106 PMC5376366 27696777

[60] Cambridge G Acharya J Cooper JA Edwards JC Humphries SE Antibodies to citrullinated peptides and risk of coronary heart disease. Atherosclerosis (2013) 228:243–6. 10.1016/j.atherosclerosis.2013.02.009 23474125

[61] Sokolove J Brennan MJ Sharpe O Lahey LJ Kao AH Krishnan E Citrullination within the atherosclerotic plaque: A potential target for the anti-citrullinated protein antibody response in rheumatoid arthritis. Arthritis Rheum (2013) 65:1719–24. 10.1002/art.37961 PMC3731137 23553485

[62] Thorp E Subramanian M Tabas I The role of macrophages and dendritic cells in the clearance of apoptotic cells in advanced atherosclerosis. Eur J Immunol (2011) 41:2515–8. 10.1002/eji.201141719 PMC3289088 21952808

[63] Blackburn JWD Lau DHC Liu EY Ellins J Vrieze AM Pawlak EN Soluble CD93 is an apoptotic cell opsonin recognized by αx β2. Eur J Immunol (2019) 49:600–10. 10.1002/eji.201847801 30656676

[64] Poteryaev D Datta S Ackema K Zerial M Spang A Identification of the switch in early-to-late endosome transition. Cell (2010) 141:497–508. 10.1016/j.cell.2010.03.011 20434987

[65] Downey GP Botelho RJ Butler JR Moltyaner Y Chien P Schreiber AD Phagosomal maturation, acidification, and inhibition of bacterial growth in nonphagocytic cells transfected with FcgammaRIIA receptors. J Biol Chem (1999) 274:28436–44. 10.1074/jbc.274.40.28436 10497205

[66] Nunes P Demaurex N Dinauer MC Regulation of the NADPH oxidase and associated ion fluxes during phagocytosis. Traffic (2013) 14:1118–31. 10.1111/tra.12115 23980663

[67] A-Gonzalez N Quintana JA García-Silva S Mazariegos M González de la Aleja A Nicolás-Ávila JA Phagocytosis imprints heterogeneity in tissue-resident macrophages. J Exp Med (2017) 214:1281–96. 10.1084/jem.20161375 PMC5413334 28432199

[68] Thorp EB Mechanisms of failed apoptotic cell clearance by phagocyte subsets in cardiovascular disease. Apoptosis (2010) 15:1124–36. 10.1007/s10495-010-0516-6 PMC3744319 20552278

[69] Thorp E Tabas I Mechanisms and consequences of efferocytosis in advanced atherosclerosis. J Leukoc Biol (2009) 86:1089–95. 10.1189/jlb.0209115 PMC2774877 19414539

[70] Zhang P Behre G Pan J Iwama A Wara-Aswapati N Radomska HS Negative cross-talk between hematopoietic regulators: GATA proteins repress PU.1. Proc Natl Acad Sci U S A (1999) 96:8705–10. 10.1073/pnas.96.15.8705 PMC17580 10411939

[71] Theilgaard-Mönch K Jacobsen LC Borup R Rasmussen T Bjerregaard MD Nielsen FC The transcriptional program of terminal granulocytic differentiation. Blood (2005) 105:1785–96. 10.1182/blood-2004-08-3346 15514007

[72] Hsu AP Sampaio EP Khan J Calvo KR Lemieux JE Patel SY Mutations in GATA2 are associated with the autosomal dominant and sporadic monocytopenia and mycobacterial infection (MonoMAC) syndrome. Blood (2011) 118:2653–5. 10.1182/blood-2011-05-356352 PMC3172785 21670465

[73] Johnson KD Hsu AP Ryu M-J Wang J Gao X Boyer ME Cis-element mutated in GATA2-dependent immunodeficiency governs hematopoiesis and vascular integrity. J Clin Invest (2012) 122:3692–704. 10.1172/JCI61623 PMC3461907 22996659

[74] Lasbury ME Tang X Durant PJ Lee C-H Effect of transcription factor GATA-2 on phagocytic activity of alveolar macrophages from Pneumocystis carinii-infected hosts. Infect Immun (2003) 71:4943–52. 10.1128/IAI.71.9.4943-4952.2003 PMC187340 12933836

[75] Tang X Lasbury ME Davidson DD Bartlett MS Smith JW Lee CH Down-regulation of GATA-2 transcription during Pneumocystis carinii infection. Infect Immun (2000) 68:4720–4. 10.1128/iai.68.8.4720-4724.2000 PMC98420 10899878

[76] Scheenstra MR Salunkhe V De Cuyper IM Hoogenboezem M Li E Kuijpers TW Characterization of hematopoietic GATA transcription factor expression in mouse and human dendritic cells. Blood Cells Mol Dis (2015) 55:293–303. 10.1016/j.bcmd.2015.07.006 26460250

[77] Menghini R Marchetti V Cardellini M Hribal ML Mauriello A Lauro D Phosphorylation of GATA2 by Akt increases adipose tissue differentiation and reduces adipose tissue-related inflammation: a novel pathway linking obesity to atherosclerosis. Circulation (2005) 111:1946–53. 10.1161/01.CIR.0000161814.02942.B2 15837948

[78] Wu T-T Tai Y-T Cherng Y-G Chen T-G Lin C-J Chen T-L GATA-2 transduces LPS-induced il-1β gene expression in macrophages via a toll-like receptor 4/MD88/MAPK-dependent mechanism. PloS One (2013) 8:e72404. 10.1371/journal.pone.0072404 23940812 PMC3735524

[79] Minegishi N Ohta J Suwabe N Nakauchi H Ishihara H Hayashi N Alternative promoters regulate transcription of the mouse GATA-2 gene. J Biol Chem (1998) 273:3625–34. 10.1074/jbc.273.6.3625 9452491

[80] Pan X Minegishi N Harigae H Yamagiwa H Minegishi M Akine Y Identification of human GATA-2 gene distal IS exon and its expression in hematopoietic stem cell fractions. J Biochem (2000) 127:105–12. 10.1093/oxfordjournals.jbchem.a022570 10731672

[81] Paul F Arkin Y Giladi A Jaitin DA Kenigsberg E Keren-Shaul H Transcriptional Heterogeneity and Lineage Commitment in Myeloid Progenitors. Cell (2015) 163:1663–77. 10.1016/j.cell.2015.11.013 26627738

[82] Korns D Frasch SC Fernandez-Boyanapalli R Henson PM Bratton DL Modulation of macrophage efferocytosis in inflammation. Front Immunol (2011) 2:57. 10.3389/fimmu.2011.00057 22566847 PMC3342042

[83] Kojima Y Volkmer J-P McKenna K Civelek M Lusis AJ Miller CL CD47-blocking antibodies restore phagocytosis and prevent atherosclerosis. Nature (2016) 536:86–90. 10.1038/nature18935 27437576 PMC4980260

[84] Lim HY Lim SY Tan CK Thiam CH Goh CC Carbajo D Hyaluronan Receptor LYVE-1-Expressing Macrophages Maintain Arterial Tone through Hyaluronan-Mediated Regulation of Smooth Muscle Cell Collagen. Immunity (2018) 49:326–341.e7. 10.1016/j.immuni.2018.06.008 30054204

[85] Moore KJ Sheedy FJ Fisher EA Macrophages in atherosclerosis: a dynamic balance. Nat Rev Immunol (2013) 13:709–21. 10.1038/nri3520 PMC4357520 23995626

[86] Wolf D Zirlik A Ley K Beyond vascular inflammation–recent advances in understanding atherosclerosis. Cell Mol Life Sci (2015) 72:3853–69. 10.1007/s00018-015-1971-6 PMC4577451 26100516

[87] Beyer M Mallmann MR Xue J Staratschek-Jox A Vorholt D Krebs W High-resolution transcriptome of human macrophages. PloS One (2012) 7:e45466. 10.1371/journal.pone.0045466 23029029 PMC3448669

[88] Martinez FO Gordon S Locati M Mantovani A Transcriptional Profiling of the Human Monocyte-to-Macrophage Differentiation and Polarization: New Molecules and Patterns of Gene Expression. J Immunol (2006) 177:7303–11. 10.4049/jimmunol.177.10.7303 17082649

