# Supplementary material for: Efferocytic Defects in Early Atherosclerosis Are Driven by GATA2 Overexpression in Macrophages
Source: Front Immunol. 2020 Oct 23;11:594136. doi: 10.3389/fimmu.2020.594136 (PMC7644460; doi:10.3389/fimmu.2020.594136)
Supplement: Supplementary file 1 [file DataSheet_1.pdf]

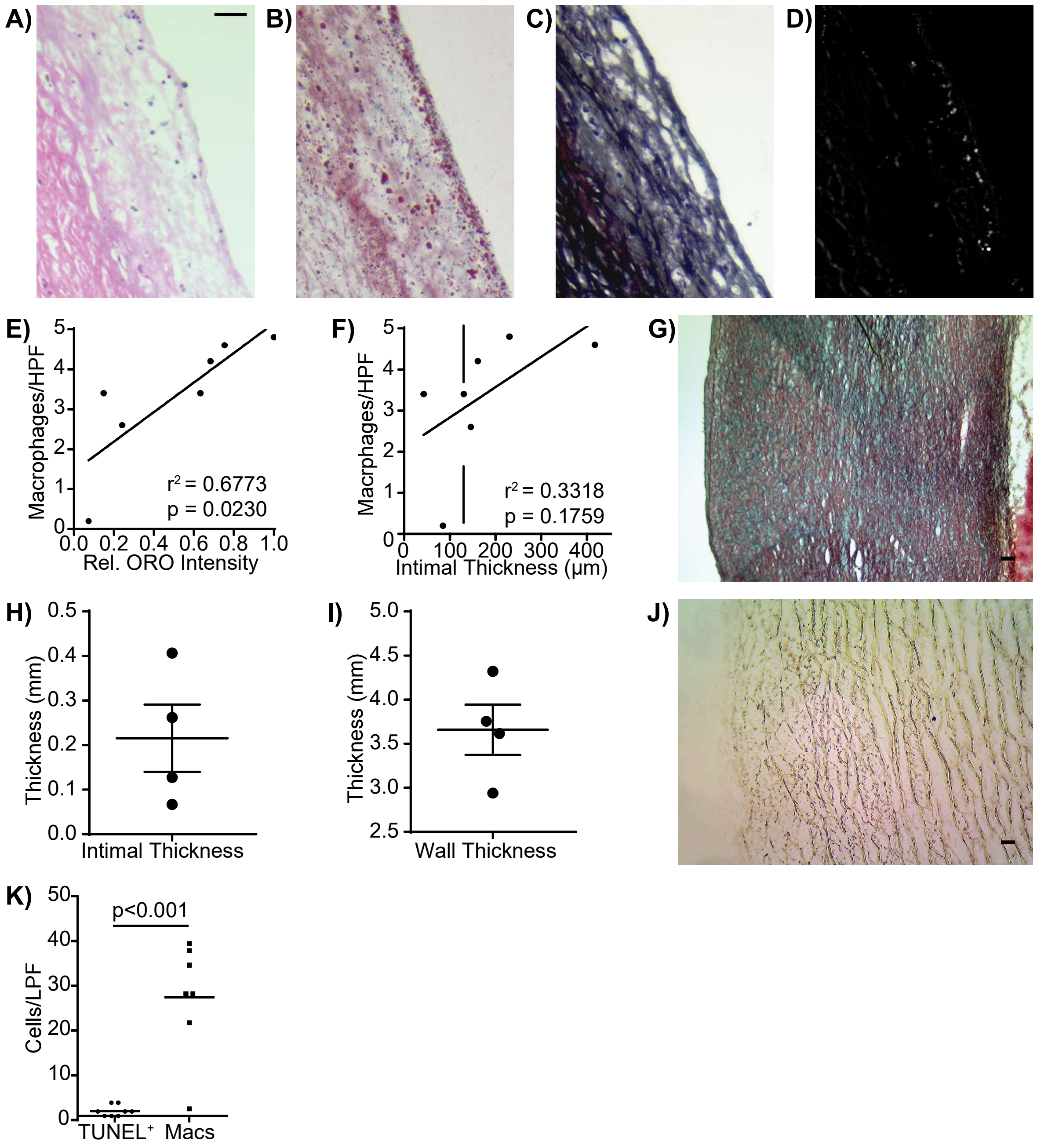

Supplemental Figure 1: Identification and Recovery of Macrophages From Early Stage Plaque. Regions of plaque development were identified using serial 10 μm sections from human aortic punch biopsies stained with **A)** H&E, **B)** Oil-Red-O (ORO), **C)** Movats pentachrome, and **D)** the macrophage marker CD163. **E)** Lipid and sterol deposition, measured by relative ORO staining, correlated strongly with macrophage numbers in high-power fields (HPF, 675 μm × 910 μm). **F)** No correlation was observed between macrophage infiltrates and intimal thickness; vertical line indicates normal aortic intimal thickness. **G-I)** Representative low-power field Movats stain (G), used to assess intimal (H) and wall (I) thickness. **J)** Representative low-power field TUNEL stain. **K)** Number of TUNEL+ cells and macrophages per low-powered field (LPF). Data are representative of, or quantifies, 4 or 5 patients or age- and sex-matched controls. Data is plotted as individual measurements plus either a linear regression (E-F) or median (H,I,K). p-values were calculated using either linear regression (E-F) or with a Mann-Whitney U test (K). Scale bars are 100 μm.

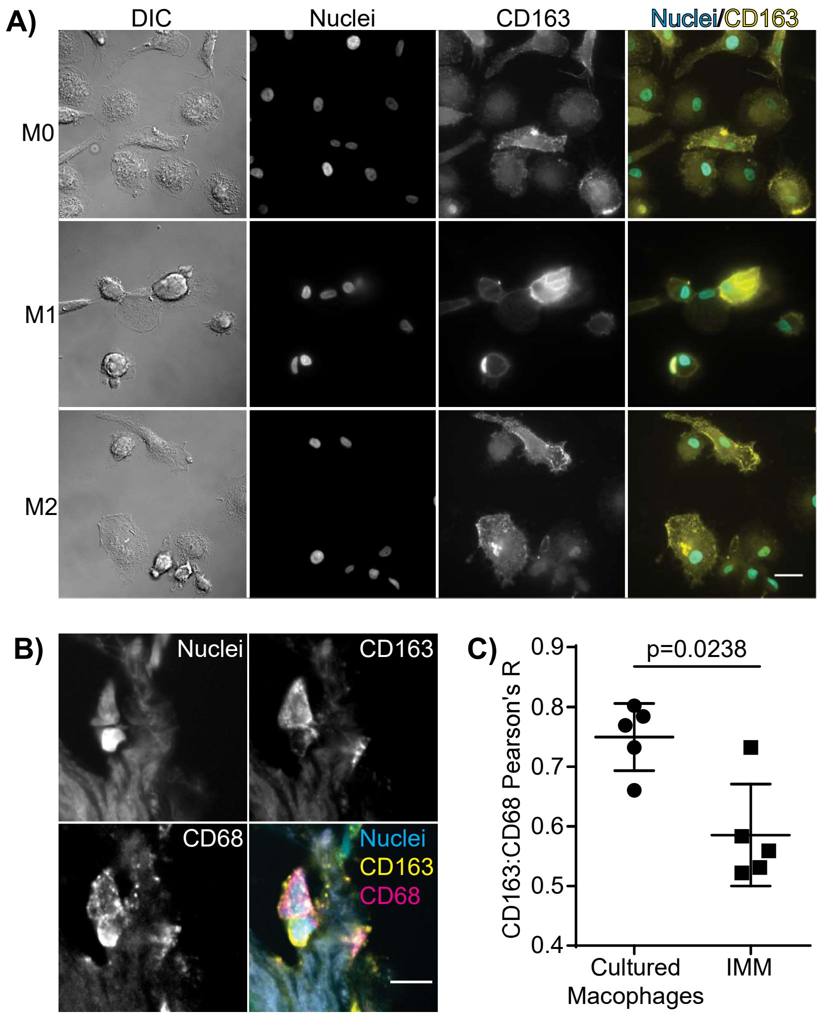

Supplemental Figure 2: Validation of CD163 as a Human Macrophage Marker. A) Monocyte-derived macrophages were polarized in vitro to M0, M1 or M2 polarization states and stained for nuclei and CD163. Scales bars are 20 µm. **B)** Nuclei (cyan), CD163 (yellow) and CD68 (magenta) staining in 10 µm frozen sections from human aortic punch biopsies confirmed to contain early-stage atherosclerotic plaque by ORO and H&E staining of serial slices (not shown). Scale bar is 10 µm. **C)** Pearson's colocalization coefficient of CD163 with CD68 in cultured macrophages and IMMs. Data are representative of, or quantifies, 5 independent experiments. p value was calculated using Mann-Whitney U-test.

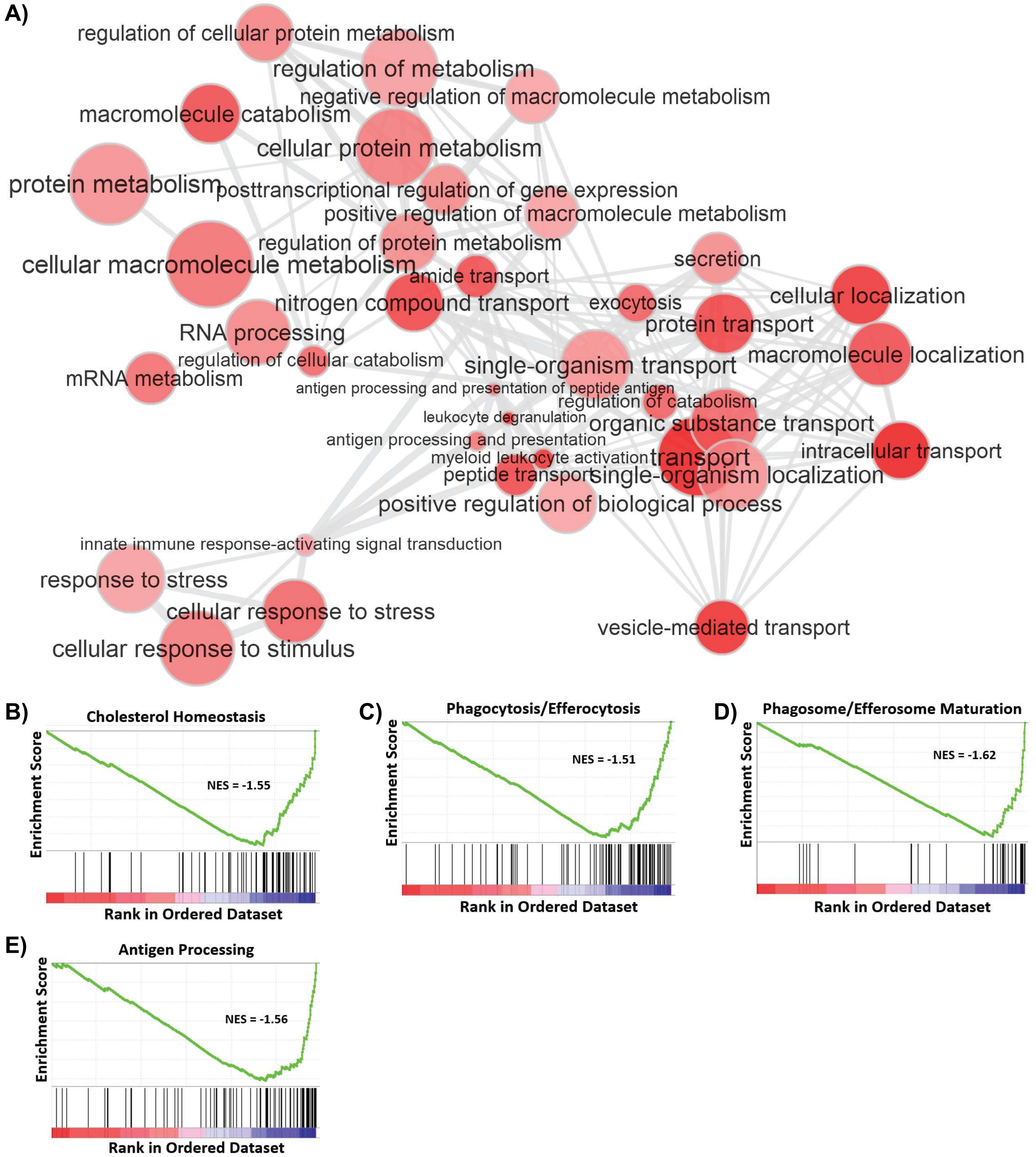

Supplementary Figure 3: Gene Ontology and Gene Set Enrichment Analysis of IIMs. A) Visualization of enriched gene ontology terms from genes differentially expressed in IIMs versus age- and sex-matched monocyte-derived macrophages, with three major clusters of differentially regulated clusters identified: (top) catabolic processes including those regulating cholesterol homeostasis, (bottom left) pathways regulating responses to cell stress, and (bottom-right) the signaling and vesicular trafficking pathways regulating phagocytosis, endocytosis and efferocytosis. **B-E)** Gene set enrichment analysis of genes differentially expressed in IIMs versus age- and sex-matched monocyte-derived macrophages, with respect to gene sets related to cholesterol homeostasis (B), engulfment of pathogens or apoptotic cells (C), degradation of phagocytosed or efferocytosed targets (D), and generation and presentation of efferosome/phagosome-derived antigens (E).

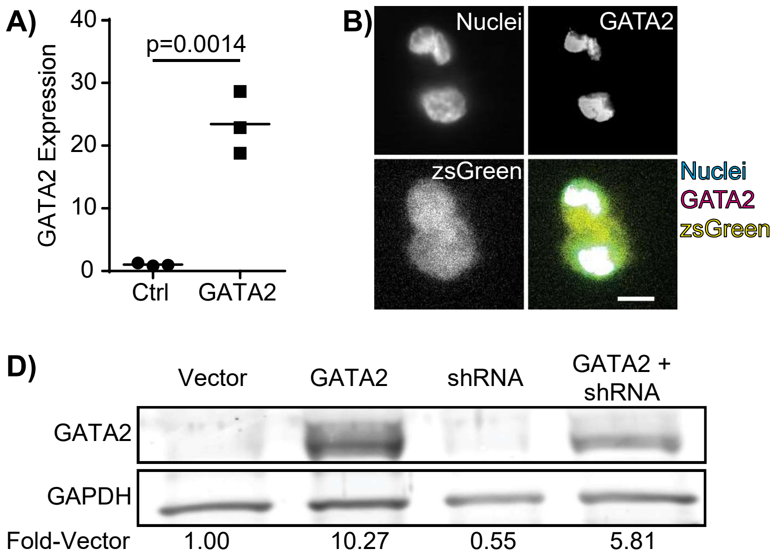

Supplemental Figure 4: Characterization of in vitro Monocyte-Derived Macrophage Models of GATA2 Perturbation. A) RT-PCR quantification of THP1 monocyte-derived macrophages expressing a GATA2 transgene (GATA2) or vector-control (Ctrl). **B)** Fluorescence imaging of representative THP1 monocyte-derived macrophages expressing a GATA2 transgene stained for nuclei and GATA2 with transfected cells also expressing a fluorescence marker (zsGreen). **C)** RT-PCR quantification of GATA2 expression in THP1 monocyte-derived macrophages expressing a GATA2-targeting shRNA (shRNA) or a scrambled shRNA control (Ctrl) following treatment with 100 µg/mL oxLDL for 72 hr. **D)** Western blot for GATA2 in THP1 mononcyte-derived macrophages expressing empty vector (Vector), a GATA2 transgene (GATA2), a GATA2-targeting shRNA (shRNA) or both a GATA2 transgene together with a GATA2-targeting shRNA (GATA2 + shRNA). Fold-change in GATA2 protein levels, normalized to GAPDH expression and the vector-control, are shown below each lane. Data are representative of (B,D), or quantify (A,C) at least 3 independent experiments. p-values were determined using Students t-test.

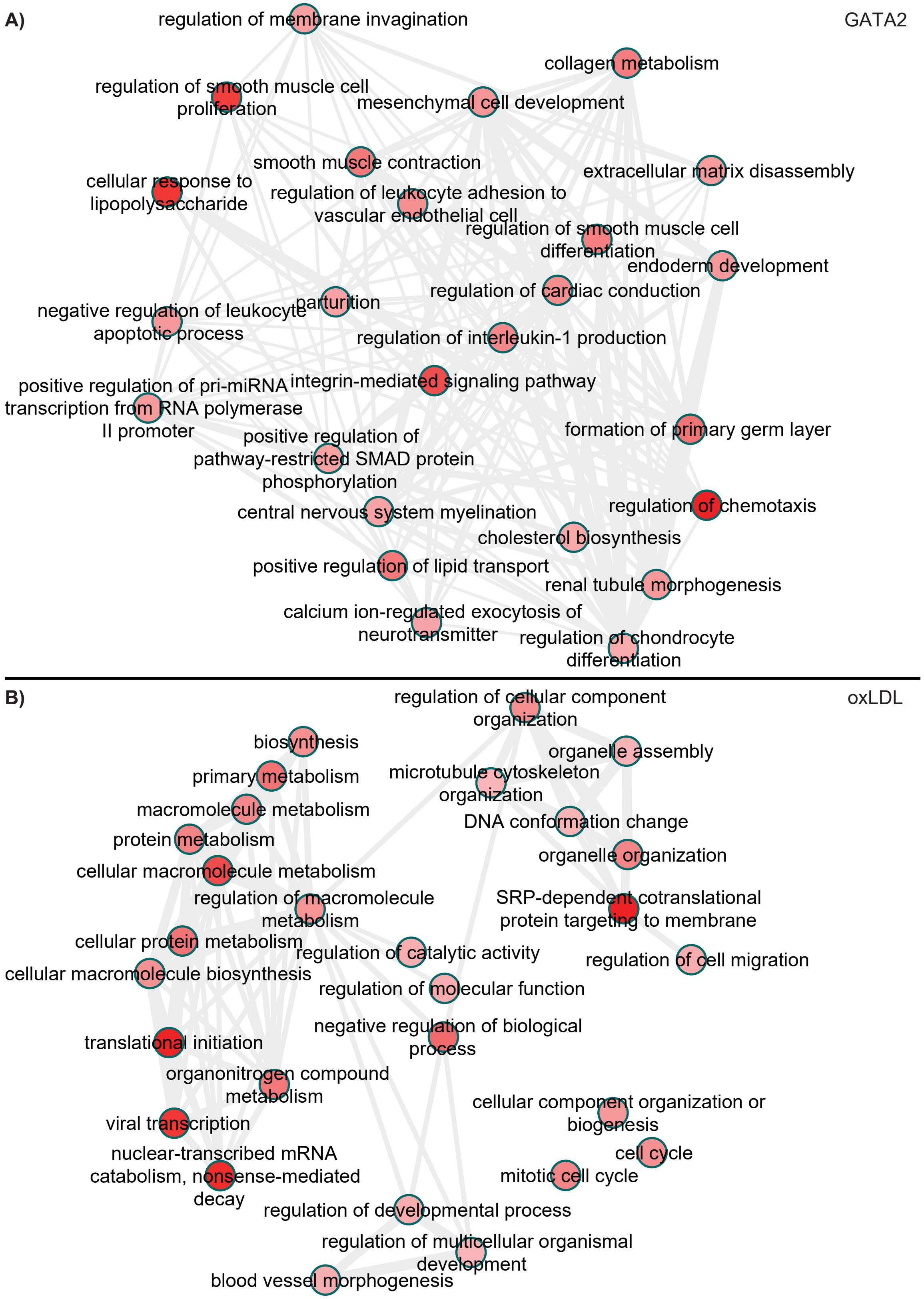

Supplemental Figure 5: Gene Ontology Analysis of GATA2-Overexpressing and oxLDL-Treated Monocyte-Derived Macrophages. A) Visualization of enriched gene ontology terms from genes differentially expressed in THP1 monocyte-derived macrophages expressing a GATA2 transgene (GATA2) compared to wild-type cells. **B)** Visualization of enriched gene ontology terms from genes differentially expressed in THP1 monocyte-derived macrophages treated with 100 µg/mL oxLDL for 72 hr (oxLDL) compared to wild-type cells.

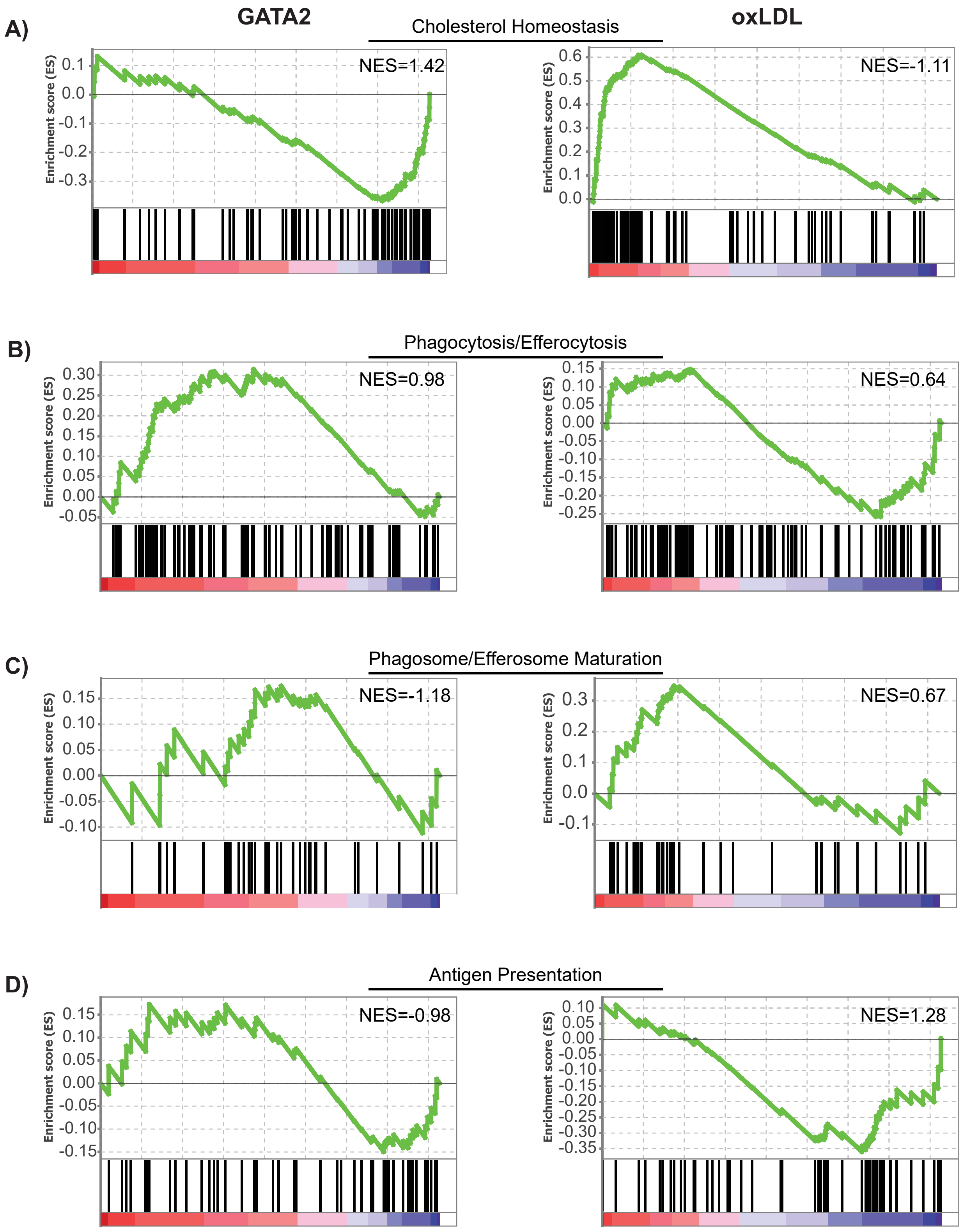

Supplemental Figure 6: Gene Set Enrichment Analysis of GATA2-Overexpressing and oxLDL-Treated Monocyte-Derived Macrophages. Gene set enrichment analysis of genes differentially expressed in THP1 monocyte-derived macrophages expressing a GATA2 transgene (GATA2) or treated with 100 µg/mL oxLDL for 72 hr (oxLDL) compared to wild-type cells. **A)** Gene sets related to cholesterol homeostasis. **B)** Gene sets related to the engulfment of pathogens or apoptotic cells. **C)** Gene sets related to the degradation of phagocytosed or efferocytosed targets. **D)** Gene sets related to the generation and presentation of efferosome/phagosome-derived antigens.

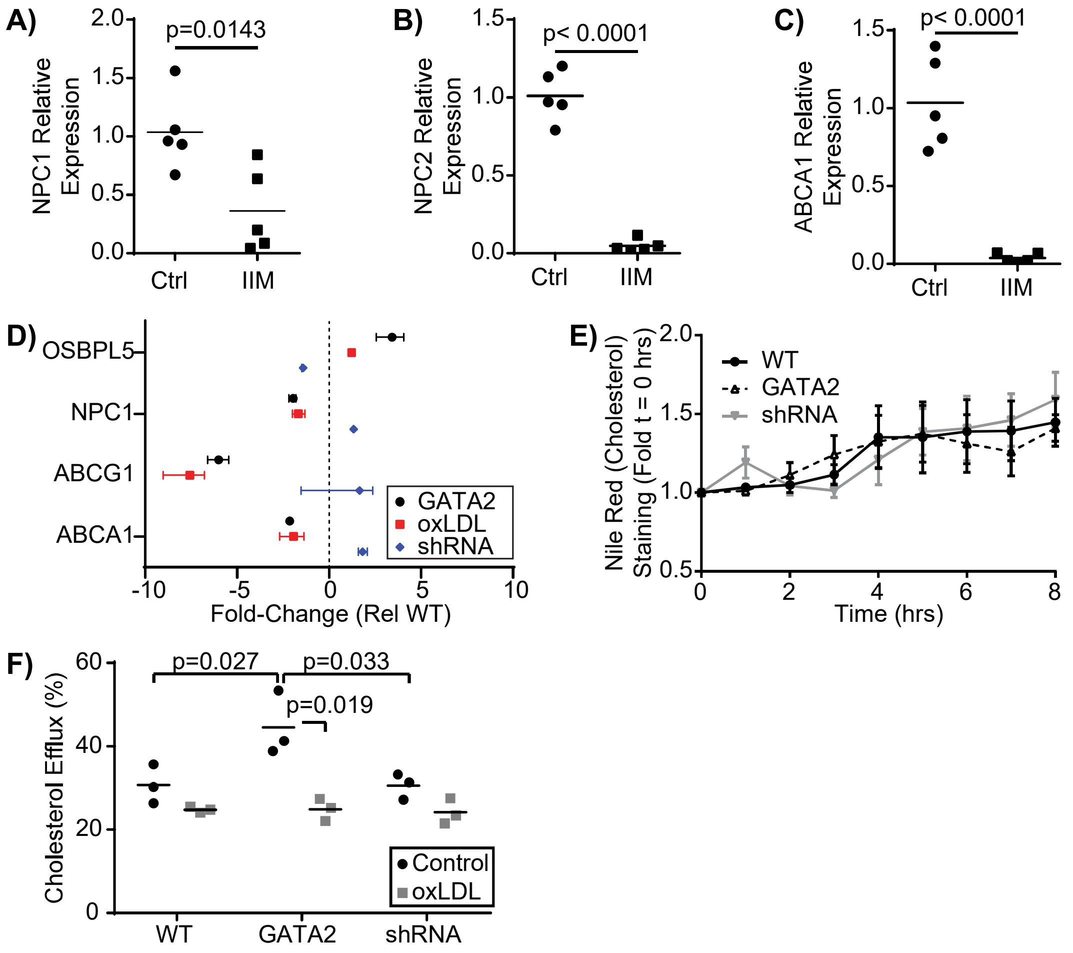

Supplemental Figure 7: Cholesterol Homeostasis is Partially Regulated by GATA2. A-C) RT-PCR quantification of the expression of cholesterol-regulating genes NPC1 (A), NPC2 (B), and ABCA1 (C) in IIMs versus age- and sex-matched monocyte-derived macrophages (Control). **D)** Changes in gene expression, compared to vector-control, of cholesterol homeostasis-associated genes in THP1 macrophages expressing a GATA2 transgene (GATA2), or treated with oxLDL ± a GATA2-targeting shRNA (oxLDL, shRNA). **E)** Quantification of intracellular lipid accumulation by Nile Red staining on THP1 monocyte-derived macrophages expressing a GATA2 transgene (GATA2), a GATA2-targeting shRNA (shRNA) or vector-control (WT), cultured with 100 µg/mL oxLDL over 8 hr. **F)** The effect of normocholesterolemia (Control) or hypercholesterolemia (oxLDL) on the efflux of fluorescently labeled cholesterol from THP1 monocyte-derived macrophages expressing a GATA2 transgene (GATA2), a GATA2-targeting shRNA (shRNA) or a vector-control (WT). Data quantify a minimum of 3 independent experiments. p-values were calculated using Students t-test (A-C) or two-way ANOVA with post-hoc Tukey correction (E-F).

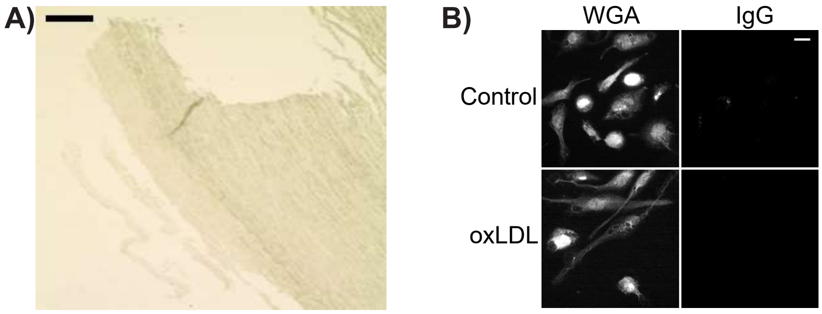

Supplemental Figure 8: Citrullination Staining Controls. A) A 10-µm section of patient aortic punch biopsy stained for citrullinated peptides. Scale bar is 10 mm. **B)** THP1 monocyte-derived macrophages that are either normocholesterolemic (Control) or hypercholesterolemic (oxLDL) stained with a plasma-membrane marker (WGA) and an IgG isotype control (IgG). Scale bar is 10 µm. Data are representative of 3 independent experiments.

Supplemental Table 1: Primer and shRNA Sequences Used in this Study.

Gene	Sequence
	qPCR primers
18S	5’-GAGGGAGCCTGAGAAACGG-3’<br>5’-GTCGGGAGTGGGTAATTTGC-3’
CD14	5’-AGCCAAGGCAGTTTGAGTCC-3’<br>5’-TAAAGGACTGCCAGCCAAGC-3’
SMA	5’-CCGACCGAATGCAGAAGGA-3’<br>5’-ACAGAGTATTTGCGCTCCG-3’
GATA2	5’-GTCACTGACGGAGAGCATGA-3’<br>5’-GGCACATAGGAGGGGTAGGT-3’
ABCA1	5’-GCACTGAGGAAGATGCTGAAA-3’<br>5’-AGTTCCTGGAAGGTCTTGTTCAC-3’
NPC1	5’-AGCCAGTAATGTCACCGAAAC-3’<br>5’-CCGAGGTTGAAGATAGTGTCG-3’
NPC2	5’-TATCCCTCTATAAAACTGGTGGTG-3’<br>5’-CCAGATGCACCGAACTCAAT-3’
RAB7A	5’-CATCCTGGGAGATTCTGGAGTC-3’<br>5’-TGTGTCCCATATCTGCATTGTG-3’
ITGAX	5’-GCTGAAGGCACACTGTGAAA-3’<br>5’-AGGGAGGCCGTGAAGTATCT-3’
ILK	5’-ATGTACTACATGAAGGCACCAATTTC-3’<br>5’-CCCCTTGCCATGTCCAAAG-3’
PADI3	5’-GGAGACCCTCGTGGACATTT-3’<br>5’-CTCCAAAGTCGCGTCAAAGC-3’
mGata2	5’-GCAGAGAAGCAAGGCTCGC-3’<br>5’-CAGTTGACACACTCCCGGC-3’
mGapdh	5’-CTCCCACTCTTCCACCTTCG-3’<br>5’-GCCTCTCTTGCTCAGTGTCC-3’
	PCR (cloning) primers
GATA2	5’-TATTTCCGGTGAATTCATGGAGGTGGCGGCCGAGCA-3’<br>5’-CGGGATCCGCGGCCGCTAGCCCATGGCGGTCACCATGCT-3’
	shRNA sequences
GATA2	5'-AAGGATCCAGCAAGGCTCGTTCCTGTTCATCAAG<br>AGTGAACAGGAACGAGCCTTGCTTTTTACCGGTAA-3'
